# Supplementary material for: Psychological inoculation strategies to fight climate disinformation across 12 countries
Source: Nat Hum Behav. 2023 Nov 30;8(2):380–98. doi: 10.1038/s41562-023-01736-0 (PMC10896732; doi:10.1038/s41562-023-01736-0)
Supplement: Supplementary file 1 — Supplementary Tables 1–14 and Figs. 1–21. [file 41562_2023_1736_MOESM1_ESM.pdf]

nature human behaviour

Registered Report

https://doi.org/10.1038/s41562-023-01736-0

Psychological inoculation strategies to fight climate disinformation across 12 countries

In the format provided by the authors and unedited

Supplementary Table of content:

1. Supplementary Table 1 – Preregistered multilevel model for affect towards climate mitigation action across conditions when processing the twenty climate disinformation statement.
2. Supplementary Figure 1: visual representation of mean affect towards climate mitigation action after the provision of twenty climate disinformation statements, across conditions.
3. Supplementary Figure 2: visual representation of mean affect towards climate mitigation action across the provision of twenty climate disinformation statements, for the scientific consensus inoculation.
4. Supplementary Figure 3: visual representation of mean affect towards climate mitigation action across the provision of twenty climate disinformation statements, for the trust in scientists inoculation.
5. Supplementary Figure 4: visual representation of mean affect towards climate mitigation action across the provision of twenty climate disinformation statements, for the transparent communications inoculation.
6. Supplementary Figure 5: visual representation of mean affect towards climate mitigation action across the provision of twenty climate disinformation statements, for the moralization inoculation.
7. Supplementary Figure 6: visual representation of mean affect towards climate mitigation action across the provision of twenty climate disinformation statements, for the accuracy inoculation.
8. Supplementary Figure 7: visual representation of mean affect towards climate mitigation action across the provision of twenty climate disinformation statements, for the positive emotions inoculation.
9. Supplementary Table 2 – Summary of psychological inoculations potential thematic match with climate disinformation statements and truth discernment items.
10. Supplementary Table 3 – Preregistered multilevel model for affect towards climate mitigation action across conditions when processing the twenty climate disinformation statement – thematic match moderation.
11. Supplementary Table 4 – Preregistered multilevel model for affect towards climate mitigation action across conditions when processing the twenty climate disinformation statement – thematic match moderation (continued).
12. Supplementary Table 5 – Preregistered multilevel models for participants' belief in the reality, anthropogenic causes, and negativity of the consequences of climate change.
13. Supplementary Table 6 – Preregistered multilevel models for WEPT performance (n. of pages completed with 90% accuracy in identifying target numbers).
14. Supplementary Table 7 – Preregistered multilevel model for truth discrimination in the climate truth discrimination task – sum score of correct responses.
15. Supplementary Figure 8: visual representation of participants' mean truth discernment in the truth discernment task, broken down by statement type, veracity, and by condition.
16. Supplementary Table 8 – Preregistered multilevel model for affect towards climate mitigation action across conditions when processing the twenty climate disinformation statement – moderation by tendency for intuitive or deliberative thinking, by pathway.
17. Supplementary Table 9 – Preregistered multilevel model for affect towards climate mitigation action across conditions when processing the twenty climate disinformation statement – moderation by tendency for intuitive or deliberative thinking, by condition.
18. Supplementary Figure 9: Visual representation of the moderation by tendency for deliberate or intuitive thinking on the scientific consensus inoculation and mean affect towards climate mitigation action across the provision of twenty climate disinformation statements.
19. Supplementary Figure 10: Visual representation of the moderation by tendency for deliberate or intuitive thinking on the trust in scientists inoculation and mean affect towards climate mitigation action across the provision of twenty climate disinformation statements.
20. Supplementary Figure 11: Visual representation of the moderation by tendency for deliberate or intuitive thinking on the transparent communications inoculation and mean affect towards climate mitigation action across the provision of twenty climate disinformation statements.

21. Supplementary Figure 12: Visual representation of the moderation by tendency for deliberate or intuitive thinking on the moralization inoculation and mean affect towards climate mitigation action across the provision of twenty climate disinformation statements.
22. Supplementary Figure 13: Visual representation of the moderation by tendency for deliberate or intuitive thinking on the accuracy inoculation and mean affect towards climate mitigation action across the provision of twenty climate disinformation statements.
23. Supplementary Figure 14: Visual representation of the moderation by tendency for deliberate or intuitive thinking on the positive emotions inoculation and mean affect towards climate mitigation action across the provision of twenty climate disinformation statements.
24. Supplementary Table 10 – Exploratory multilevel model for affect towards climate mitigation action across conditions when processing the twenty climate disinformation statement – moderation by political ideology.
25. Supplementary Figure 15: Visual representation of the moderation by political ideology on the passive disinformation control condition and affect towards climate mitigation action across the provision of twenty climate disinformation statements.
26. Supplementary Figure 16: Visual representation of the moderation by political ideology on the scientific consensus inoculation and affect towards climate mitigation action across the provision of twenty climate disinformation statements.
27. Supplementary Figure 17: Visual representation of the moderation by political ideology on the trust in scientists inoculation and affect towards climate mitigation action across the provision of twenty climate disinformation statements.
28. Supplementary Figure 18: Visual representation of the moderation by political ideology on the moralization inoculation and affect towards climate mitigation action across the provision of twenty climate disinformation statements.
29. Supplementary Figure 19: Visual representation of the moderation by political ideology on the transparent communications inoculation and affect towards climate mitigation action across the provision of twenty climate disinformation statements.
30. Supplementary Figure 20: Visual representation of the moderation by political ideology on the accuracy inoculation and affect towards climate mitigation action across the provision of twenty climate disinformation statements.
31. Supplementary Figure 21: Visual representation of the moderation by political ideology on the positive emotions inoculation and affect towards climate mitigation action across the provision of twenty climate disinformation statements.
32. Supplementary Table 11 – Exploratory multilevel models for participants' belief in the reality, anthropogenic causes, and negativity of the consequences of climate change – moderation by political ideology.
33. Supplementary Table 12 – Exploratory multilevel model for WEPT performance and climate truth discrimination – moderation by political ideology.
34. Supplementary Table 13 – Preregistered multilevel model for affect towards climate mitigation action across conditions when processing the twenty climate disinformation statement – moderation by demand effects on psychological inoculations.
35. Supplementary Table 14 – Preregistered multilevel model for affect towards climate mitigation action across conditions when processing the twenty climate disinformation statement – moderation by demand effects.

Supplementary Table 1 – Preregistered multilevel model for affect towards climate mitigation action across conditions when processing the twenty climate disinformation statement.

Predictor	Estimate	SE	t-value	95% Confidence Intervals		P
				Lower	Upper	
Intercept	65.00	2.05	31.651	60.98	69.03	<.001
Age	-0.05	0.02	-2.602	-0.09	-0.01	.009
Gender		F-value(3, 5938):	5.6080			<.001
Political ideology	-0.22	0.11	-2.013	-0.43	-0.01	.04
Trial	-0.21	0.02	-10.559	-0.25	-0.17	<.001
Condition		F-value(6, 6978):	1.9400			.07
Condition: Scientific consensus	2.76	1.08	2.550	0.64	4.87	.01
Condition: Trust in scientists	2.28	1.07	2.130	0.18	4.38	.03
Condition: Transparent communications	1.00	1.07	0.928	-1.11	3.10	.35
Condition: Moralization	2.68	1.06	2.517	0.59	4.76	.01
Condition: Accuracy	1.16	1.07	1.080	-0.95	3.27	.28
Condition: Positive emotions	2.05	1.07	2.339	0.41	4.61	.02
Trial * Condition		F-value(6, 112994):	1.4749			.18
Trial * Condition: Scientific consensus	-0.04	0.03	-1.493	-0.01	0.01	.14
Trial * Condition: Trust in scientists	-0.03	0.03	-0.976	-0.08	0.03	.33
Trial * Condition: Transparent communications	-0.06	0.03	-1.943	-0.11	0.0005	.052
Trial * Condition: Moralization	0.003	0.03	-0.114	-0.06	0.05	0.91
Trial * Condition: Accuracy	-0.06	0.03	-1.985	-0.11	-0.001	.047

Trial * Condition: Positive emotions	-0.01	0.03	-0.280	-0.07	0.05	.78

Note: condition contrast codes are in reference to the passive control condition. Two-sided tests, α corrected to .005 for condition contrasts.

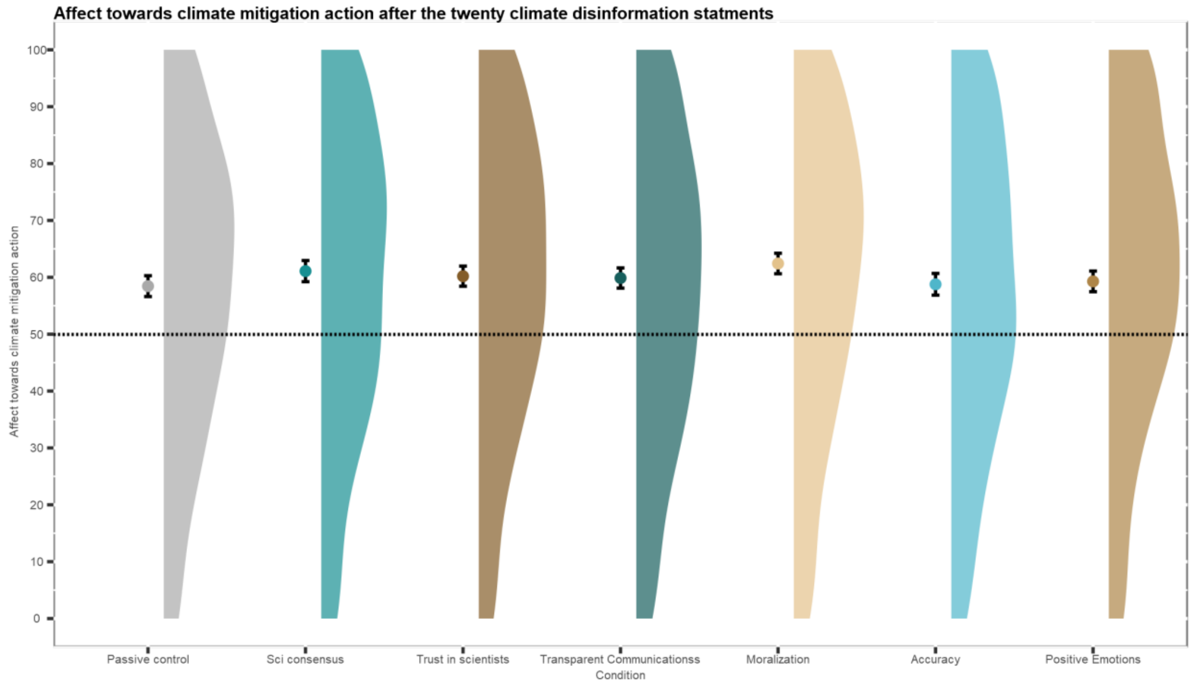

Supplementary Figure 1: Visual representation of mean affect towards climate mitigation action after the provision of twenty climate disinformation statements, across conditions. The x axis represents the experimental conditions, in order from the bottom: pure control condition (light gray); passive control condition (dark gray); scientific consensus inoculation (light green); trust in scientists inoculation (dark brown); transparent communications inoculation (dark green); moralization inoculation (gold); accuracy inoculation (light blue); and positive emotions inoculation (light brown). Error bars represent the 95% confidence intervals. Color palette by MetBrewer package. The y axis represents mean affect towards climate mitigation action, with values increasing from 50 related to feeling overall more positively towards climate mitigation action, and values decreasing below 50 related to feeling overall more negatively towards climate mitigation action. The dashed line represents the "*neutral*" anchor point (Affect=50) in the visual analog scale. Color palette by MetBrewer package. Error bars represent the mean-centered 95% confidence intervals. Passive disinformation control condition: two-sided t-test: $t(1676.83)=-6.774$, $p<.001$, $\delta=-0.33$, 95% CI[-10.48, -5.77]. Scientific consensus inoculation: equivalence test: $t(1667.05)=-3.239$, $p<.001$, 90% CI[-1.46, 3.72]. Trust in scientists inoculation: equivalence test: $t(1705.36)=3.272$, $p<.001$, 90% CI[-1.02, 3.69]. Transparent communication inoculation: equivalence test: $t(1685.78)=-3.769$, $p<.001$, 90% CI[-1.03, 3.29]. Moralization inoculation: equivalence test: $t(1730)=-2.630$, $p=.004$, 90% CI[-0.15, 4.12]. Accuracy inoculation: equivalence test: $t(1683.24)=3.960$, $p<.001$, 90% CI[-2.36, 1.97]. Positive emotions inoculation: equivalence test: $t(1702.68)=-2.243$, $p=.012$, 95% CI[0.28, 4.54].

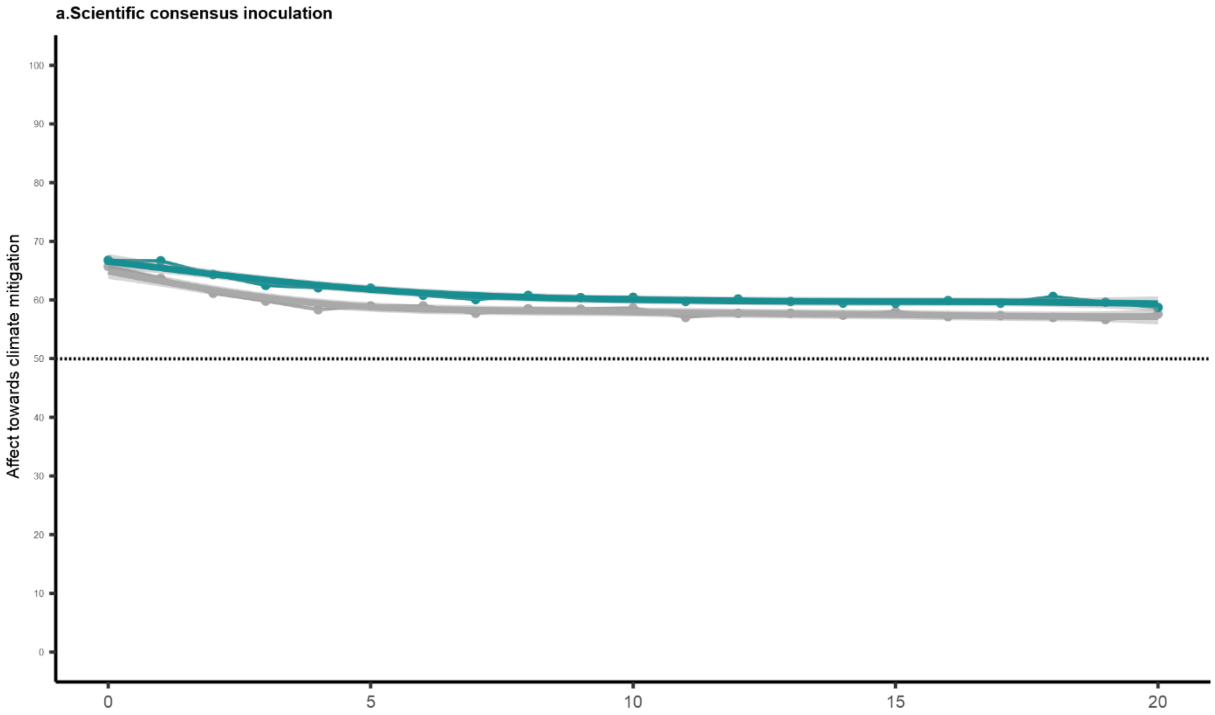

Supplementary Figure 2: Visual representation of mean affect towards climate mitigation action across the provision of twenty climate disinformation statements, for the scientific consensus inoculation and its contrast with the passive control condition (represented in dark gray). The y axis represents mean affect towards climate mitigation action, with values increasing from 50 related to feeling overall more positively towards climate mitigation action, and values decreasing below 50 related to feeling overall more negatively towards climate mitigation action. The dashed line represents the "*neutral*" anchor point (Affect=50) in the visual analog scale. The x axis represents the trial number, with Trial=0 representing affect pre-intervention, and the numbers 1 to 20 representing each climate disinformation statement received. Light gray bands represent the mean-centered standard errors produced by model fitting with a GAM function. Color palette by MetBrewer package. Contrast between conditions: $t_{\text{two-sided}}$ (6978)=2.550, p=.01, β=2.78, 95% CI[0.64, 4.87]. Two-way interaction between condition and trial: $t_{\text{two-sided}}$ (113000)=-1.493, p=.14, β=-0.04, 95% CI[-0.01, 0.01].

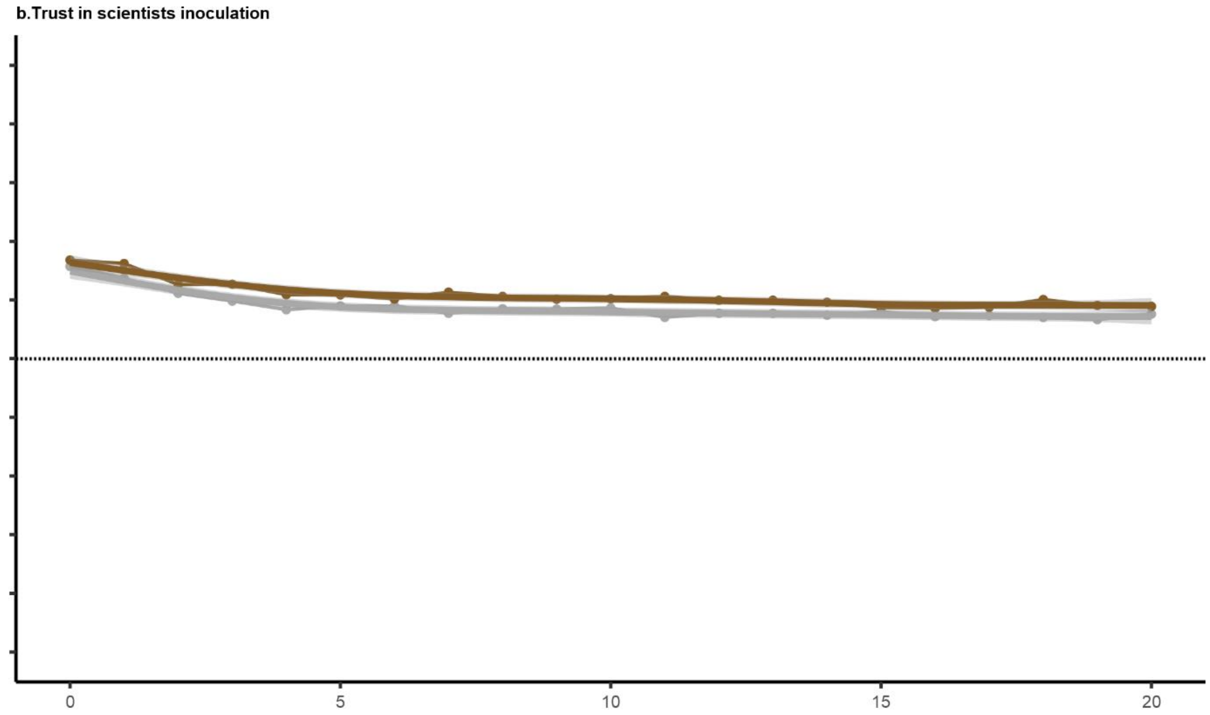

Supplementary Figure 3: Visual representation of mean affect towards climate mitigation action across the provision of twenty climate disinformation statements, for the trust in scientists inoculation and its contrast with the passive control condition (represented in dark gray). The y axis represents mean affect towards climate mitigation action, with values increasing from 50 related to feeling overall more positively towards climate mitigation action, and values decreasing below 50 related to feeling overall more negatively towards climate mitigation action. The dashed line represents the "*neutral*" anchor point (Affect=50) in the visual analog scale. The x axis represents the trial number, with Trial=0 representing affect pre-intervention, and the numbers 1 to 20 representing each climate disinformation statement received. Light gray bands represent the mean-centered standard errors produced by model fitting with a GAM function. Color palette by MetBrewer package. Contrast between conditions: $t_{\text{two-sided}}$ (6978)=2.130, p=.033, β=2.28, 95% CI[0.18, 4.38]. Two-way interaction between condition and trial: $t_{\text{two-sided}}$ (113000)=-0.976, p=.33, β=-0.03, 95% CI[-0.08, 0.03].

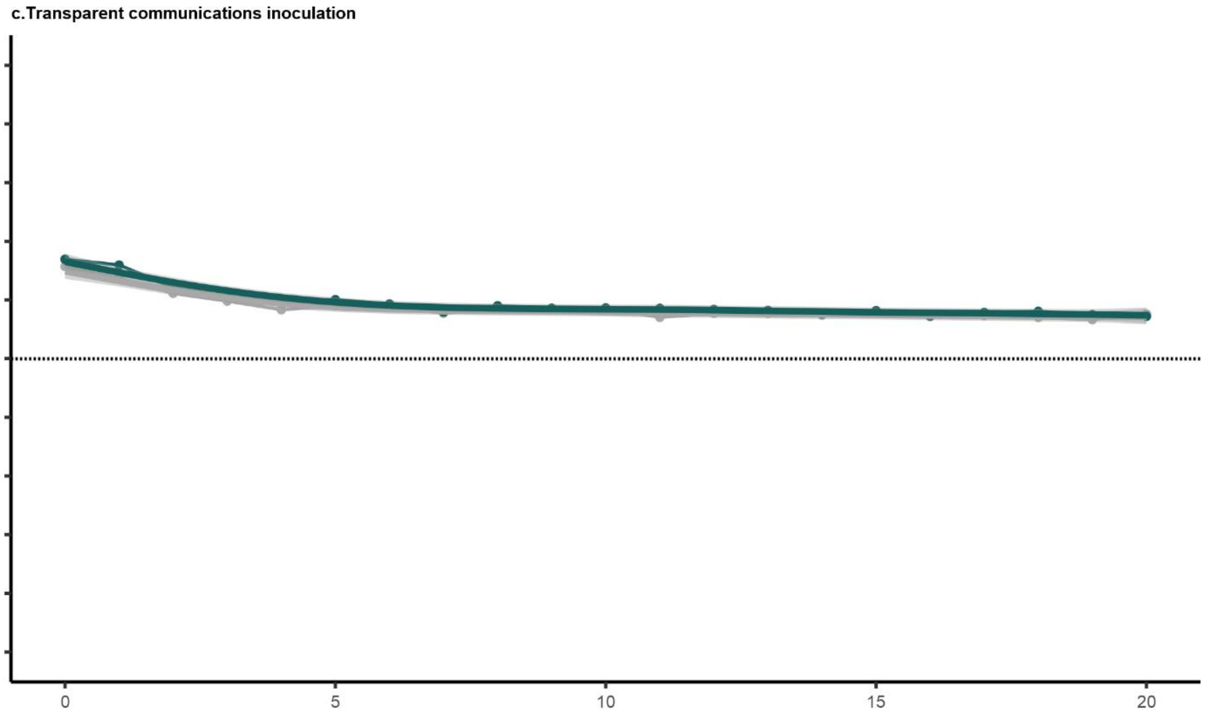

Supplementary Figure 4: Visual representation of mean affect towards climate mitigation action across the provision of twenty climate disinformation statements, for the transparent communications inoculation and its contrast with the passive control condition (represented in dark gray). The y axis represents mean affect towards climate mitigation action, with values increasing from 50 related to feeling overall more positively towards climate mitigation action, and values decreasing below 50 related to feeling overall more negatively towards climate mitigation action. The dashed line represents the "*neutral*" anchor point (Affect=50) in the visual analog scale. The x axis represents the trial number, with Trial=0 representing affect pre-intervention, and the numbers 1 to 20 representing each climate disinformation statement received. Light gray bands represent the mean-centered standard errors produced by model fitting with a GAM function. Color palette by MetBrewer package. Contrast between conditions: $t_{\text{two-sided}}(6978)=0.928$, $p=.35$, $\beta=1.00$, 95% CI[-1.11, 3.10]. Two-way interaction between condition and trial: $t_{\text{two-sided}}(113000)=-1.943$, $p=.052$, $\beta=-0.06$, 95% CI[-0.11, 0.0005].

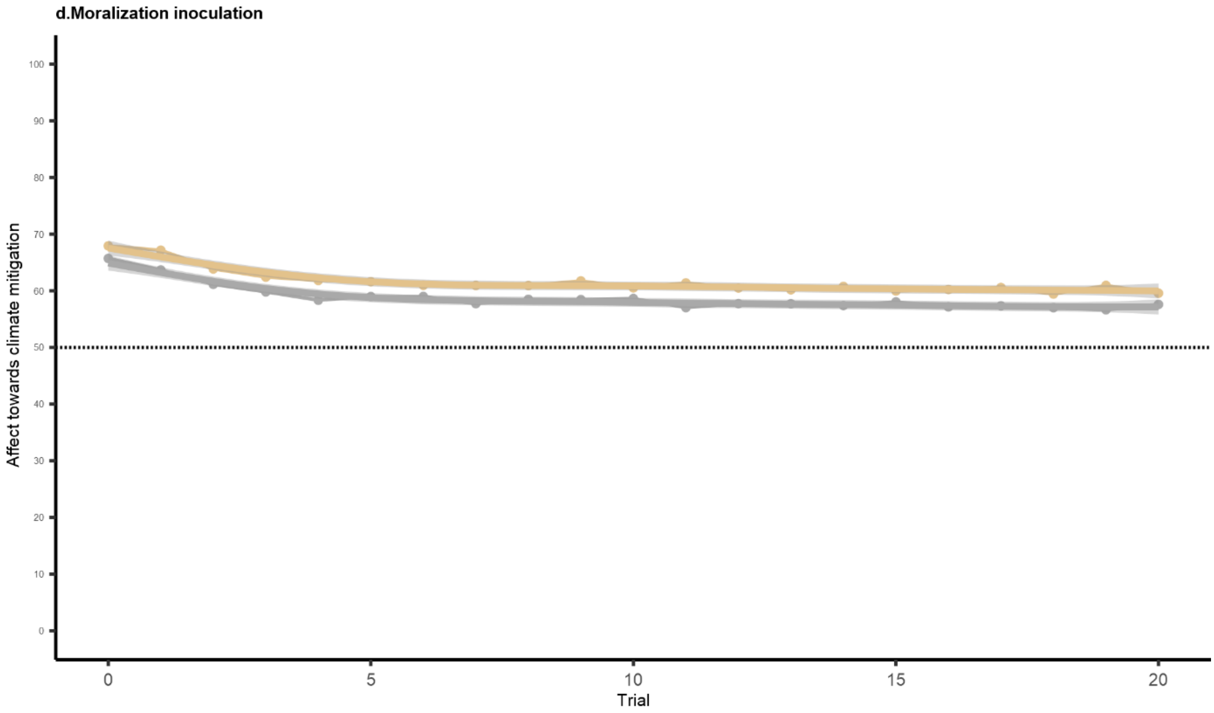

Supplementary Figure 5: Visual representation of mean affect towards climate mitigation action across the provision of twenty climate disinformation statements, for the moralization inoculation and its contrast with the passive control condition (represented in dark gray). The y axis represents mean affect towards climate mitigation action, with values increasing from 50 related to feeling overall more positively towards climate mitigation action, and values decreasing below 50 related to feeling overall more negatively towards climate mitigation action. The dashed line represents the "*neutral*" anchor point (Affect=50) in the visual analog scale. The x axis represents the trial number, with Trial=0 representing affect pre-intervention, and the numbers 1 to 20 representing each climate disinformation statement received. Light gray bands represent the mean-centered standard errors produced by model fitting with a GAM function. Color palette by MetBrewer package. Contrast between conditions: $t_{\text{two-sided}}(6978)=2.517$, p=.011, β=2.68, 95% CI[0.59, 4.76]. Two-way interaction between condition and trial: $t_{\text{two-sided}}(113000)=-0.114$, p=.91, β=-0.003, 95% CI[-0.06, 0.05].

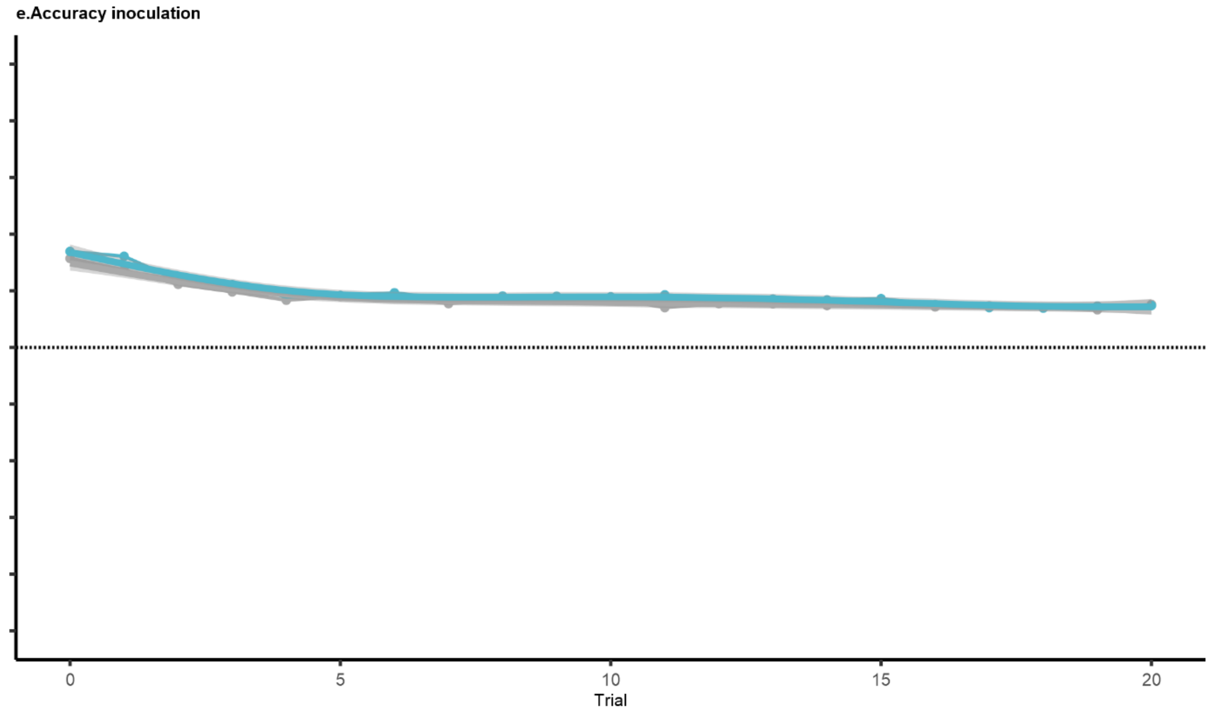

Supplementary Figure 6: Visual representation of mean affect towards climate mitigation action across the provision of twenty climate disinformation statements, for the accuracy inoculation and its contrast with the passive control condition (represented in dark gray). The y axis represents mean affect towards climate mitigation action, with values increasing from 50 related to feeling overall more positively towards climate mitigation action, and values decreasing below 50 related to feeling overall more negatively towards climate mitigation action. The dashed line represents the "*neutral*" anchor point (Affect=50) in the visual analog scale. The x axis represents the trial number, with Trial=0 representing affect pre-intervention, and the numbers 1 to 20 representing each climate disinformation statement received. Light gray bands represent the mean-centered standard errors produced by model fitting with a GAM function. Color palette by MetBrewer package. Contrast between conditions: $t_{\text{two-sided}}(6978)=1.080$, p=.033, β=1.16, 95% CI[-0.95, 3.27]. Two-way interaction between condition and trial: $t_{\text{two-sided}}(113000)=-1.985$, p=.047, β=-0.06, 95% CI[-0.11, -0.001].

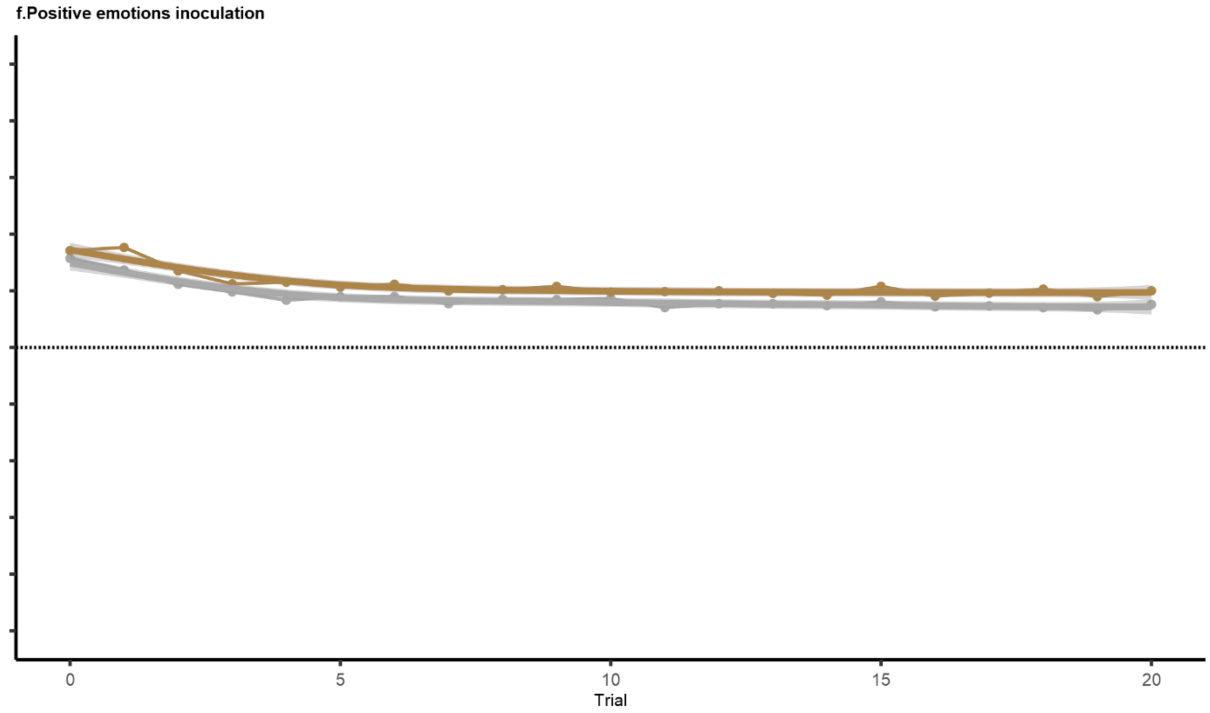

Supplementary Figure 7: Visual representation of mean affect towards climate mitigation action across the provision of twenty climate disinformation statements, for the positive emotions inoculation and its contrast with the passive control condition (represented in dark gray). The y axis represents mean affect towards climate mitigation action, with values increasing from 50 related to feeling overall more positively towards climate mitigation action, and values decreasing below 50 related to feeling overall more negatively towards climate mitigation action. The dashed line represents the "*neutral*" anchor point (Affect=50) in the visual analog scale. The x axis represents the trial number, with Trial=0 representing affect pre-intervention, and the numbers 1 to 20 representing each climate disinformation statement received. Light gray bands represent the mean-centered standard errors produced by model fitting with a GAM function. Color palette by MetBrewer package[**Error! Reference source not found.**]. Contrast between conditions: $t_{\text{two-sided}}$ (6978)=2.339, p=.02, β=0.01, 95% CI[0.41, 4.61]. Two-way interaction between condition and trial: $t_{\text{two-sided}}$ (113000)=-0.280, p=.78, β=-0.01, 95% CI[-0.06, 0.05].

Supplementary Table 2 – Summary of psychological inoculations potential thematic match with climate disinformation statements and truth discernment items.

Inoculation	Matching climate disinformation statement(s)
Scientific consensus	Science_8; Science_10
Trust in scientists	Science_4; Science_5; Science_6
Transparent communication	Action_8; Action_10
Moralization of climate action	Action_4; Action_5; Action_9
Accuracy	
Positive emotion	

Supplementary Table 3 – Preregistered multilevel model for affect towards climate mitigation action across conditions when processing the twenty climate disinformation statement – thematic match moderation.

	Thematic match with the scientific consensus inoculation						Thematic match with the trust in scientists inoculation					
				95% Confidence Intervals						95% Confidence Intervals		
	Estimate	SE	t-value	Lower	Upper	p	Estimate	SE	t-value	Lower	Upper	p
Intercept	64.27	2.55	25.224	59.27	69.26	<.001	61.96	2.71	22.905	56.66	67.26	<.001
Age	-0.07	0.04	-1.801	-0.15	0.01	.07	-0.01	0.04	-0.272	-0.08	0.06	.79
Gender	F-value (3, 1669.2):		1.2770			.28	F-value (3, 1704):		0.9794			.40
Political ideology	0.003	0.20	0.018	-0.39	0.39	.99	-0.13	0.20	-0.686	-0.52	0.25	.49
Trial	-0.22	0.04	-5.814	-0.28	-0.14	<.001	-0.21	0.03	-6.134	-0.28	-0.14	<.001
Condition	2.89	1.08	2.678	0.77	5.01	.007	2.07	1.07	1.929	-0.03	4.16	.054
Thematic Match	0.86	1.14	-0.296	-1.38	3.10	.46	-0.96	1.00	-0.963	-2.91	0.99	.35
Thematic Match * Condition	F-value (1, 31000.5):		1.9041			.17	F-value (1, 31649):		1.8532			.17
Trial * Condition	-0.04	0.05	-0.826	-1.80	0.31	.41	-0.03	0.05	-0.568	-0.12	0.07	.57

Note: condition contrast codes are in reference to the passive control condition. Two-sided tests, α=.05. Thematic match contrast codes refer to climate disinformation statements unmatching versus matching thematically with the psychological inoculation of interest.

Supplementary Table 4 – Preregistered multilevel model for affect towards climate mitigation action across conditions when processing the twenty climate disinformation statement – thematic match moderation (continued).

	Thematic match with the transparent communications inoculation						Thematic match with the moralization inoculation					
				95% Confidence Intervals						95% Confidence Intervals		
	Estimate	SE	t-value	Lower	Upper	p	Estimate	SE	t-value	Lower	Upper	p
Intercept	64.55	2.59	24.910	59.47	69.63	<.001	63.38	2.56	24.752	58.36	68.40	<.001
Age	-0.08	0.04	-1.941	-0.15	0.001	.052	-0.01	0.04	-0.340	-0.09	0.06	.73
Gender	F-value (3, 1690.8):		1.6037			.19	F-value (3, 1723):		0.5651			.64
Political ideology	0.06	0.19	-0.314	-0.44	0.32	.75	-0.30	0.20	-1.497	-0.68	0.09	.13
Trial	-0.22	0.04	-5.814	-0.28	-0.15	<.001	-0.21	0.03	-6.198	-0.28	-0.15	<.001
Condition	1.08	1.06	1.010	-1.01	3.16	.31	2.81	1.07	2.633	0.72	4.91	.009
Thematic Match	0.06	1.22	0.046	-2.33	2.45	.96	2.32	0.99	0.234	-1.71	2.17	.82
Thematic Match * Condition	F-value (1, 31543.3):		2.4227			.12	F-value (1, 32132):		3.6287			.057
Trial * Condition	-0.05	0.05	-1.133	-0.15	0.22	.26	-0.03	0.05	-0.568	-0.10	0.09	.95

Note: Condition contrast codes are in reference to the passive control condition. Two-sided tests, α=.05. Thematic match contrast codes refer to climate disinformation statements unmatching versus matching thematically with the psychological inoculation of interest.

Supplementary Table 5 – Preregistered multilevel models for participants' belief in the reality, anthropogenic causes, and negativity of the consequences of climate change.

	Belief in the reality of climate change						Belief in the anthropogenic causes of climate change						Belief in the negativity of the consequences of climate change					
				95% Confidence Intervals						95% Confidence Intervals						95% Confidence Intervals		
Predictor	Estimate	SE	t-value	Lower	Upper	P	Estimate	SE	t-value	Lower	Upper	p	Estimate	SE	t-value	Lower	Upper	p
Intercept	4.26	0.10	52.7704	4.10	4.41	<.001	4.24	0.09	48.240	4.06	4.41	<.001	4.20	0.08	54.327	4.05	4.36	<.001
Age	0.001	0.001	0.747	-0.001	0.003	.45	-0.003	0.001	-2.853	-0.005	-0.001	.004	-0.001	0.001	-1.101	-0.003	0.001	.31

Gender	F-value(3, 5939.5):		3.0891			.026	F-value(3, 5938.8):		4.7922			.002	F-value(3, 5939.4):		5.6363			<.001
Political ideology	-0.05	0.005	-10.579	-0.06	-0.04	<.001	-0.04	0.01	-7.001	-0.05	-0.03	<.001	-0.04	0.005	-7.779	-0.05	-0.03	<.001
Condition	F-value(6, 5936.1):			1.1185		.35	F-value(6, 5936.1):		0.3236			.92	F-value(6, 5936.1):			0.2808		.94
Condition: Scientific consensus	0.08	0.05	1.584	-0.02	0.17	.11	-0.04	0.05	-0.910	-0.14	0.05	.36	-0.01	0.05	-0.201	-0.10	0.08	.84
Condition: Trust in scientists	0.02	0.05	0.416	-.007	0.11	.68	0.002	0.05	0.040	-0.09	0.10	.97	-0.01	0.05	-0.317	-0.10	0.07	.75
Condition: Transparent commu-nications	0.06	0.05	1.203	-0.04	0.15	.23	0.01	0.05	0.221	-0.08	0.11	.82	-0.01	0.05	-0.230	-0.10	0.08	.82
Condition: Morali-zation	010	0.05	2.068	0.005	0.19	.039	0.004	0.05	0.086	-0.09	0.10	.93	0.04	0.05	0.788	-0.05	0.12	.43
Condition: Accuracy	0.05	0.05	0.973	-0.05	0.14	.33	-0.001	0.05	-0.014	-0.10	0.09	.99	-0.004	0.05	-0.108	-0.09	0.08	.91
Condition: Positive emotions	0.9	0.05	1.802	-0.01	0.18	.07	-0.02	0.05	-0.510	-0.12	0.07	.61	-0.004	0.05	-0.091	-0.09	0.08	.93

Note: Condition contrast codes are in reference to the passive control condition. Two-sided tests, α corrected to .005 for condition contrasts.

Supplementary Table 6 – Preregistered multilevel models for WEPT performance (n. of pages completed with 90% accuracy in identifying target numbers).

	WEPT (Poisson)						WEPT (Poisson, zero-inflation)						WEPT (linear)					
				95% Confidence Intervals						95% Confidence Intervals						95% Confidence Intervals		
Predictor	Estimate	SE	z-value	Lower	Upper	p	Estimate	SE	z-value	Lower	Upper	p	Estimate	SE	t-value	Lower	Upper	p
Intercept	-0.40	0.08	-4.754	-0.56	-0.23	<.001	0.52	0.07	7.514	0.38	0.65	<.001	0.33	0.17	1.977	0.003	0.65	.052

Age	0.02	0.001	26.230	0.018	0.022	<.001	0.01	0.001	16.408	0.01	0.02	<.001	0.03	0.002	15.020	0.03	0.04	<.001
Gender	X(3):		33.3 04			$.6^{e-07}$	X(3):		13.5267			.004	F-value(3, 5940.8):		3.3816			0.028
Political ideology	-0.02	0.004	-4.005	-0.02	-0.01	$.6^{e-05}$	-0.01	0.005	-1.74	0.001	10.82	.08	-0.02	0.02	-2.197	-0.13	-0.003	.03
Condition	X(6):		17.074			.009	X(6):		11.7805			.07	F-value(6, 5936.2):		0.9125			.48
Condition: Scientific consensus	0.04	0.04	1.080	-0.03	0.12	.28	-3.20	4.92	-0.07	0.10	6.45	.70	0.07	0.11	0.695	-0.13	0.28	.49
Condition: Trust in scientists	-0.07	0.04	-1.646	-0.14	0.01	.10	-0.18	1.87	-0.19	-0.02	3.48	.021	-0.09	0.10	-0.862	-0.29	0.11	.39
Condition: Transparent commu-nications	0.04	0.04	1.064	-0.04	0.12	.29	6.13	4.54	-0.12	0.05	15.03	.37	0.07	0.10	0.664	-0.14	0.27	.51
Condition: Morali-zation	0.002	0.04	0.052	-0.07	0.08	.96	3.30	2.57	-0.13	0.04	8.34	.28	0.01	0.10	0.110	-0.19	0.21	.91
Condition: Accuracy	-0.03	0.04	-0.671	-0.10	0.05	.50	0.81	2.73	-0.15	0.02	6.16	.11	-0.03	0.11	-0.297	-0.24	0.17	.77
Condition: Positive emotions	0.07	0.04	1.850	-0.004	0.15	.06	-1.22	1.56	-0.08	0.08	1.83	.97	0.01	0.10	1.125	-0.09	0.32	.26
Zero-inflated Intercept							-0.12	0.02	-4.02	-0.17	-0.06	<.001						

Note: Condition contrast codes are in reference to the passive control condition. Two-sided tests, α corrected to .005 for condition contrasts.

Supplementary Table 7 – Preregistered multilevel model for truth discrimination in the climate truth discrimination task – sum score of correct responses.

				95% Confidence Intervals		
Predictor	Estimate	SE	t-value	Lower	Upper	P

Intercept	0.11	0.16	68.366	10.64	11.26	<.001
Age	0.03	0.002	12.484	0.02	0.03	<.001
Gender		F-value(3, 5941.7):	2.5752			.052
Political ideology	-0.9	.001	-8.501	-0.12	-0.07	<.001
Condition		F-value(6, 5936.4):	2.4338			.024
Condition: Scientific consensus	0.04	0.11	0.11	0.400	-0.17	0.25
Condition: Trust in scientists	0.19	0.11	1.830	-0.01	0.40	.07
Condition: Transparent communications	0.17	0.11	1.584	-0.04	0.40	.11
Condition: Moralization	0.09	0.11	0.878	-0.11	0.30	.38
Condition: Accuracy	0.36	0.11	3.360	0.15	0.57	<.001
Condition: Positive emotions	0.11	0.11	1.063	-0.09	0.32	.29

Note: Condition contrast codes are in reference to the passive control condition. Two-sided tests, α corrected to .005 for condition contrasts.

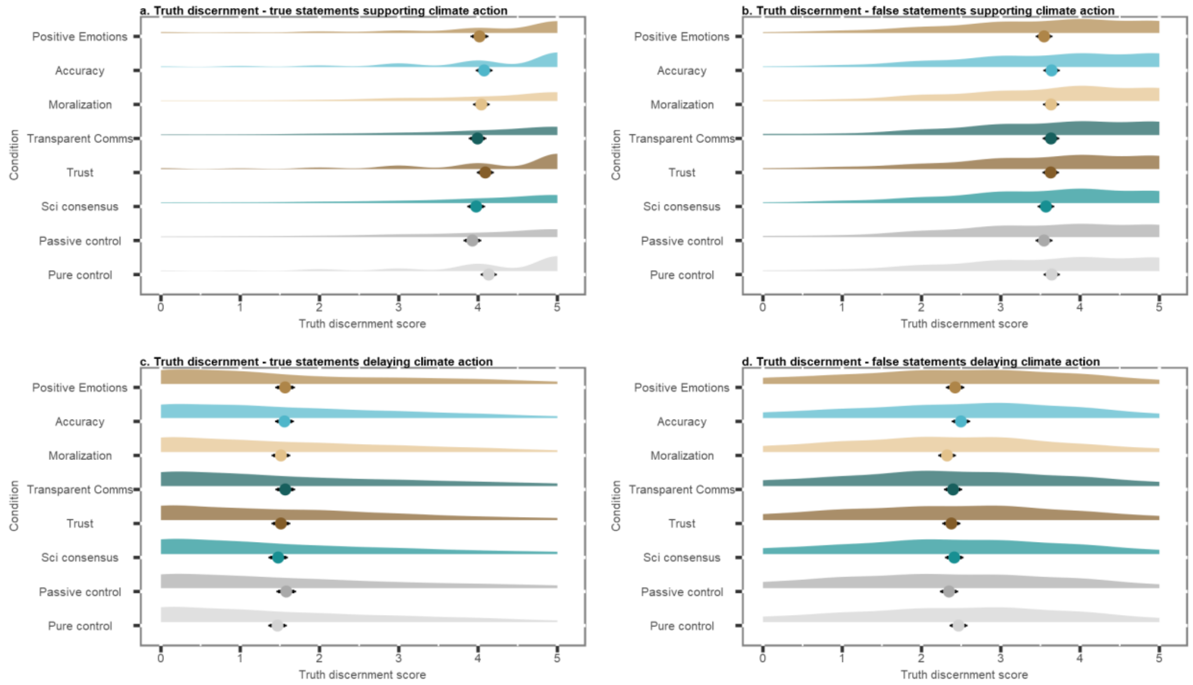

Supplementary Figure 8: Discernment by statement type and veracity. The y axis represents the experimental conditions. The x axis represents mean correct responses. Error bars represent mean-centered 95% confidence intervals. Color palette by MetBrewer. Panel a: Mean discernment score, true statements supporting climate action. Pure control: two-sided t-test: $t(1685.96)=3.312$, $p<.001$, 95% CI[0.08, 0.33]. Scientific consensus inoculation: equivalence test: $t(1710.75)=-3.410$, $p=.003$, 90% CI[-0.08, 0.17]. Trust in scientists inoculation: two-sided t-test: $t(1705.11)=2.556$, $p=.011$, 95% CI[0.04, 0.29]. Transparent communications inoculation: equivalence test: $t(1710.74)=-3.144$, $p<.001$, 90% CI[-0.04, 0.17]. Moralization inoculation: equivalence test: $t(1739.88)=-2.376$, $p=.009$, 90% CI[0.01, 0.21]. Accuracy inoculation: equivalence test: $t(1743.59)=-2.690$, $p=.004$, 90% CI[-0.01, 0.19]. Positive emotions inoculation: equivalence test: $t(1744.98)=-2.807$, $p=.014$, 90% CI[-0.02, 0.20]. Panel b: Mean discernment score, false statements supporting climate action. Passive disinformation control: equivalence test: $t(1705.82)=-2.531$, $p=.006$, 90% CI[-0.002, 0.19]. Scientific consensus inoculation: equivalence test: $t(1706.83)=2.205$, $p<.001$, 90% CI[-0.07, 0.12]. Trust in scientists inoculation: equivalence test: $t(1709.2)=-2.743$, $p=.003$, 90% CI[-0.01, 0.18]. Transparent communications inoculation: equivalence test: $t(1710.58)=-2.698$, $p=.004$, 90% CI[-0.01, 0.19]. Moralization inoculation: equivalence test: $t(1705.67)=-2.664$, $p=.004$, 90% CI[-0.01, 0.19]. Accuracy inoculation: equivalence test: $t(1704.82)=-2.549$, $p=.005$, 90% CI[-0.003, 0.19]. Positive emotions inoculation: equivalence test: $t(1710.11)=-4.130$, $p<.001$, 90% CI[-0.01, 0.01]. Panel c: Mean discernment score, true statements delaying climate action. Passive disinformation control: equivalence test: $t(1699.49)=-2.600$, $p=.005$, 90% CI[-0.01, 0.23]. Scientific consensus inoculation:

equivalence test: $t(1707.82)=-2.737$, $p=.003$, 90% CI[-0.02, 0.22]. Trust in scientists inoculation: equivalence test: $t(1702.96)=-3.203$, $p<.001$, 90% CI[-0.05, 0.19]. Transparent communications inoculation: equivalence test: $t(1712.1)=-3.973$, $p<.001$, 90% CI[-0.11, 0.13]. Moralization inoculation: equivalence test: $t(1734.94)=-3.207$, $p<.001$, 90% CI[-0.05, 0.19]. Accuracy inoculation: equivalence test: $t(1699.83)=-3.827$, $p<.001$, 90% CI[-0.10, 0.14]. Positive emotions inoculation: equivalence test: $t(1711.88)=-3.944$, $p<.001$, 90% CI[-0.11, 0.14]. Panel d: Mean discernment score, false statements delaying climate action. Passive disinformation control: equivalence test: $t(1711.07)=2.356$, $p=.009$, 90% CI[-0.23, -0.01]. Scientific consensus inoculation: equivalence test: $t(1711.99)=3.166$, $p<.001$, 90% CI[-0.18, 0.04]. Trust in scientists inoculation: equivalence test: $t(1712.06)=3.708$, $p<.001$, 90% CI[-0.14, 0.086]. Transparent communications inoculation: equivalence test: $t(1711.31)=3.383$, $p<.001$, 90% CI[-0.16, 0.06]. Moralization inoculation: equivalence test: $t(1709.08)=-3.764$, $p<.001$, 90% CI[-0.08, 0.13]. Accuracy inoculation: two-sided t-test: $t(1743.74)=2.204$, $p=.024$, $\delta=0.11$, 95% CI[0.02, 0.27]. Positive emotions inoculation: equivalence test: $t(1712.79)=3.310$, $p=.001$, 90% CI[-0.19, 0.03].

Supplementary Table 8 – Preregistered multilevel model for affect towards climate mitigation action across conditions when processing the twenty climate disinformation statement – moderation by tendency for intuitive or deliberative thinking, by pathway.

				95% Confidence Intervals		
Predictor	Estimate	SE	t-value	Lower	Upper	P
Intercept	69.06	2.25	30.705	64.65	73.47	<.001
Age	-0.07	0.02	-2.974	-1.11	-0.02	.003
Gender		F-value(3, 5085):	6.7105			<.001
Political ideology	-0.28	0.12	-2.438	-0.51	-0.06	.015
Trial	-0.33	0.03	-11.355	-3.84	-0.27	<.001
CRT-2 score	-1.37	0.64	-2.135	-2.62	-0.11	.033
Pathway		F-value(1,5964):	0.2652			.61
Socioaffective pathway	0.25	0.49	0.515	-7.03	1.20	.61
Trial*CRT-2 score	0.03	0.02	1.971	-0.0002	0.07	.049
Trial * Pathway	F-value(1,96787):		9.7819			.002
Trial* Socioaffective pathway	0.04	0.01	3,128	0.01	0.07	.002
CRT-2 score * Pathway	F-value(1,5963):		0.2634			.61
CRT-2 score* Socioaffective pathway	0.15	0.28	0.513	-0.41	0.70	.61
Trial * CRT-2 score * Pathway	F-value(1,96787):		4.4144			.036
Trial * CRT-2 score * Socioaffective pathway	-0.02	0.01	-2.101	-0.03	-0.001	.036

Note: Condition contrast codes are in reference to the cognitive pathway (aggregated scientific consensus, transparent communications, and accuracy inoculations). Two-sided tests, α=.05.

Supplementary Table 9 – Preregistered multilevel model for affect towards climate mitigation action across conditions when processing the twenty climate disinformation statement – moderation by tendency for intuitive or deliberative thinking, by condition.

				95% Confidence Intervals		
Predictor	Estimate	SE	t-value	Lower	Upper	P
Intercept	68.15	2.31	29.479	64.65	73.47	<.001

Age	-0.07	0.02	-2.969	-1.11	-0.02	.003
Gender		F-value(3, 5085):	6.5110			.002
Political ideology	-0.29	0.12	-2.257	-0.51	-0.06	.012
Trial	-0.21	0.03	-6.369	-3.84	-0.27	<.001
CRT-2 score	0.54	0.70	0.767	-2.62	-0.11	.44
Condition		F-value(1,5965):	0.7533			.58
Trial*CRT-2 score	0.03	0.02	1.971	-0.0002	0.07	.049
Trial * Condition		F-value(1,96787):	4.0517			.001
CRT-2 score * Condition		F-value(1,5965):	2.7063			.02
Trial * CRT-2 score * Condition		F-value(1,96787):	4.0823			.001

Note: Condition contrast codes are in reference to the passive control condition. Two-sided tests, α corrected to .005 for condition contrasts.

Decomposing the CRT-2 score and condition two and three way interactions highlighted a main effect of the tendency for deliberate thinking for the participants inoculated with the transparent communications inoculation (*F-ratio*=10.254, *p*=.001) and positive emotions inoculation (*F-ratio*=6.507, *p*=.011), but not for the participants inoculated with the scientific consensus inoculation (*F-ratio*=0.047, *p*=.83), the trust inoculation (*F-ratio*=3.279, *p*=.07), the moralization inoculation (*F-ratio*=0.236, *p*=.63), and the accuracy inoculation (*F-ratio*=2.859, *p*=.09). The trial-by-trial difference in the protective effects of the inoculations was not significantly moderated by CRT-2 scores for the positive emotions inoculation (*F-ratio*=0.172, *p*=.68) and for the moralization inoculation (*F-ratio*=0.508, *p*=.48).

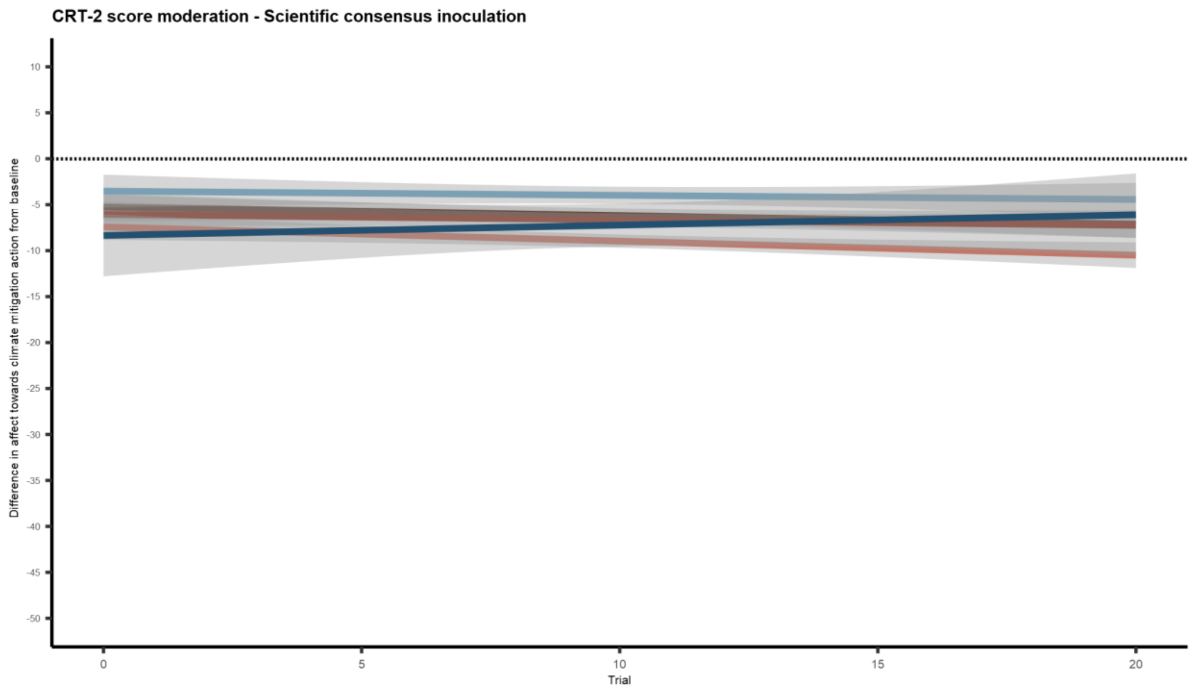

Supplementary Figure 9: Visual representation of the moderation by tendency for deliberate or intuitive thinking on the scientific consensus inoculation and mean affect towards climate mitigation action across the provision of twenty climate disinformation statements. Each color represents each level of tendency for deliberate thinking (CRT-2 score of 3 in represented light blue, CRT-2 score of 4 represented in dark blue) and intuitive thinking (CRT-2 score of 0 represented in dark red, CRT-2 score of 1 represented in red). The y axis represents the mean difference affect towards climate mitigation action from baseline (pre-inoculation and pre-disinformation provision), to better visualize the different slopes of CRT-2 scores, within condition and across trials. Values increasing from 0 are related to feeling overall more positively towards climate mitigation action, and values decreasing below 0 related to feeling overall more negatively towards climate mitigation action. The dashed line represents no mean difference from baseline. The x axis represents the trial number, with Trial=0 representing affect pre-intervention, and the numbers 1 to 20 representing each climate disinformation statement received. Light gray bands represent the mean-centered 95% CI produced by fitting a linear model. Color palette by MetBrewer package. Simple slope for CRT-2 scores: F_{ratio} (1, Inf)=0.047, p=.83. Simple slope of the two-way interaction between CRT-2 scores and trial: F_{ratio} (1, Inf)=4.087, p=.043.

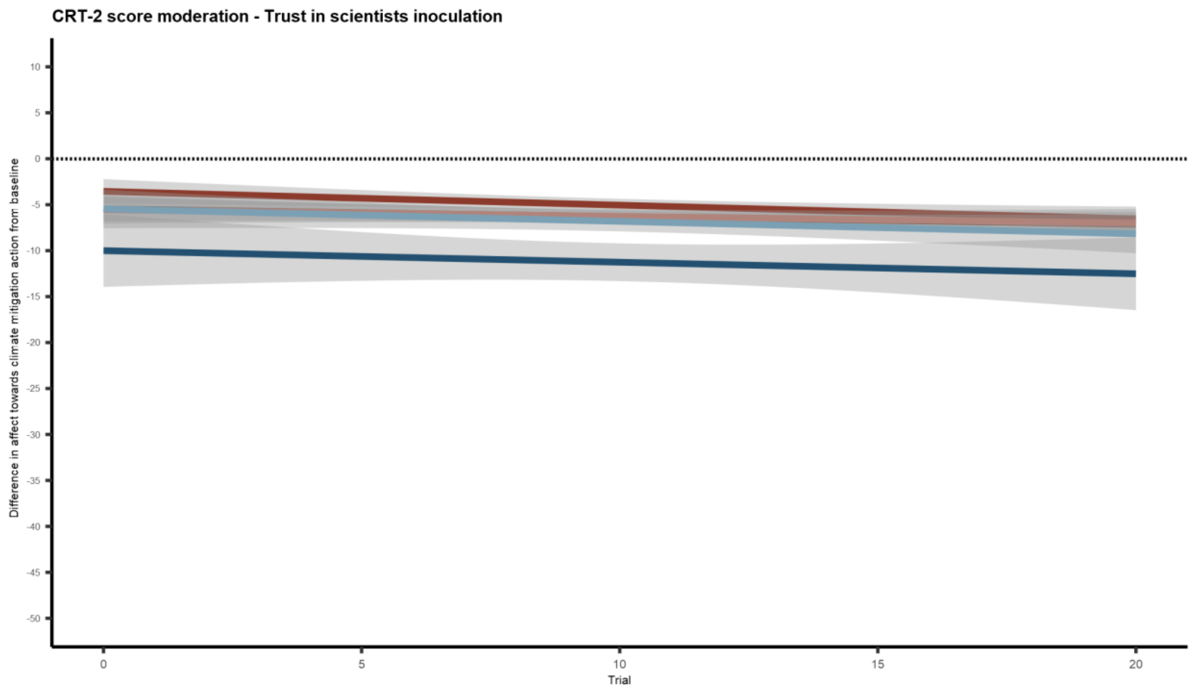

Supplementary Figure 10: Visual representation of the moderation by tendency for deliberate or intuitive thinking on the trust in scientists inoculation and mean affect towards climate mitigation action across the provision of twenty climate disinformation statements. Each color represents each level of tendency for deliberate thinking (CRT-2 score of 3 in represented light blue, CRT-2 score of 4 represented in dark blue) and intuitive thinking (CRT-2 score of 0 represented in dark red, CRT-2 score of 1 represented in red). The y axis represents the mean difference affect towards climate mitigation action from baseline (pre-inoculation and pre-disinformation provision), to better visualize the different slopes of CRT-2 scores, within condition and across trials. Values increasing from 0 are related to feeling overall more positively towards climate mitigation action, and values decreasing below 0 related to feeling overall more negatively towards climate mitigation action. The dashed line represents no mean difference from baseline. The x axis represents the trial number, with Trial=0 representing affect pre-intervention, and the numbers 1 to 20 representing each climate disinformation statement received. Light gray bands represent the mean-centered 95% CI produced by fitting a linear model. Color palette by MetBrewer package. Simple slope for CRT-2 scores: F_{ratio} (1, Inf)=3,279, p=.07. Simple slope of the two-way interaction between CRT-2 scores and trial: F_{ratio} (1, Inf)=4.102, p=.043.

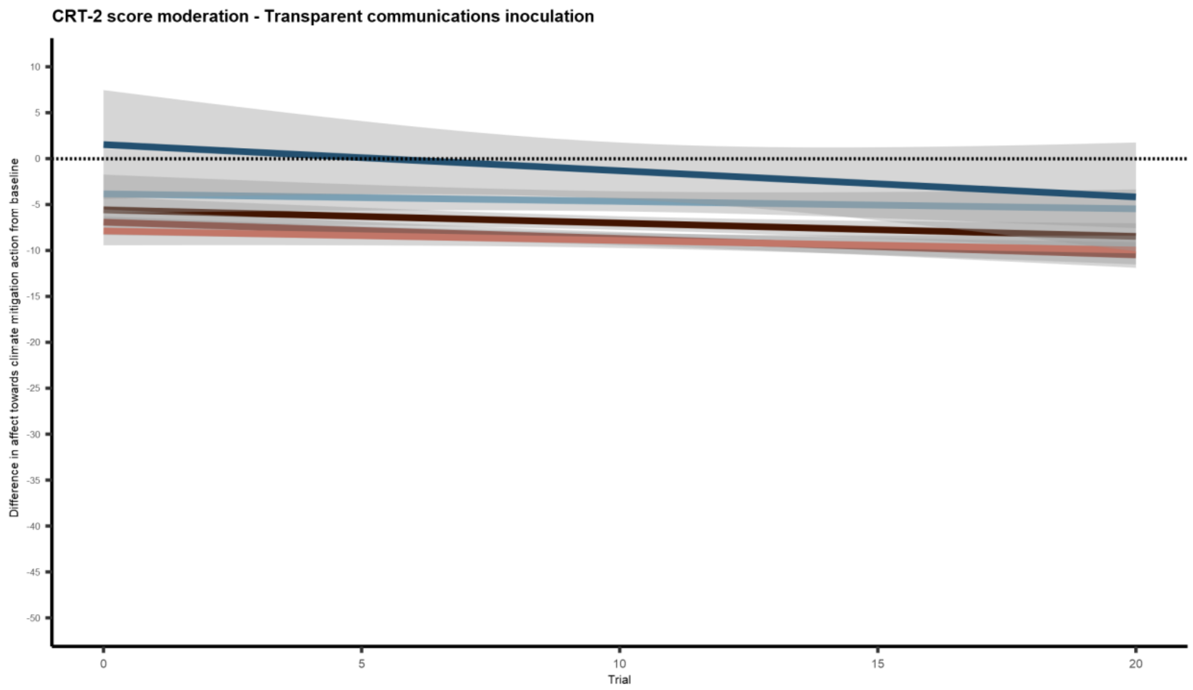

Supplementary Figure 11: Visual representation of the moderation by tendency for deliberate or intuitive thinking on the transparent communications inoculation and mean affect towards climate mitigation action across the provision of twenty climate disinformation statements. Each color represents each level of tendency for deliberate thinking (CRT-2 score of 3 in represented light blue, CRT-2 score of 4 represented in dark blue) and intuitive thinking (CRT-2 score of 0 represented in dark red, CRT-2 score of 1 represented in red). The y axis represents the mean difference affect towards climate mitigation action from baseline (pre-inoculation and pre-disinformation provision), to better visualize the different slopes of CRT-2 scores, within condition and across trials. Values increasing from 0 are related to feeling overall more positively towards climate mitigation action, and values decreasing below 0 related to feeling overall more negatively towards climate mitigation action. The dashed line represents no mean difference from baseline. The x axis represents the trial number, with Trial=0 representing affect pre-intervention, and the numbers 1 to 20 representing each climate disinformation statement received. Light gray bands represent the mean-centered 95% CI produced by fitting a linear model. Color palette by MetBrewer package. Simple slope for CRT-2 scores: F_{ratio} (1, Inf)=10.254, p=.001. Simple slope of the two-way interaction between CRT-2 scores and trial: F_{ratio} (1, Inf)=6.265, p=.012.

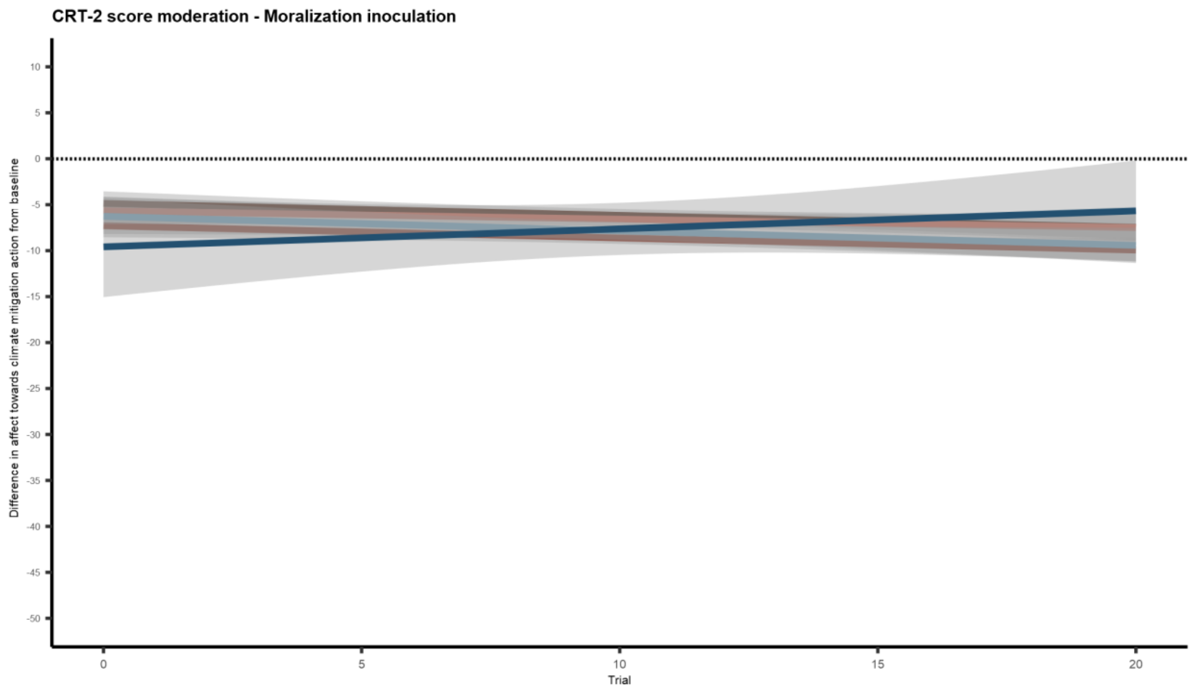

Supplementary Figure 12: Visual representation of the moderation by tendency for deliberate or intuitive thinking on the moralization inoculation and mean affect towards climate mitigation action across the provision of twenty climate disinformation statements. Each color represents each level of tendency for deliberate thinking (CRT-2 score of 3 in represented light blue, CRT-2 score of 4 represented in dark blue) and intuitive thinking (CRT-2 score of 0 represented in dark red, CRT-2 score of 1 represented in red). The y axis represents the mean difference affect towards climate mitigation action from baseline (pre-inoculation and pre-disinformation provision), to better visualize the different slopes of CRT-2 scores, within condition and across trials. Values increasing from 0 are related to feeling overall more positively towards climate mitigation action, and values decreasing below 0 related to feeling overall more negatively towards climate mitigation action. The dashed line represents no mean difference from baseline. The x axis represents the trial number, with Trial=0 representing affect pre-intervention, and the numbers 1 to 20 representing each climate disinformation statement received. Light gray bands represent the mean-centered 95% CI produced by fitting a linear model. Color palette by MetBrewer package. Simple slope for CRT-2 scores: F_{ratio} (6978)=0.236, p=.63. Simple slope of the two-way interaction between CRT-2 scores and trial: F_{ratio} (1, Inf)=0.508, p=.48.

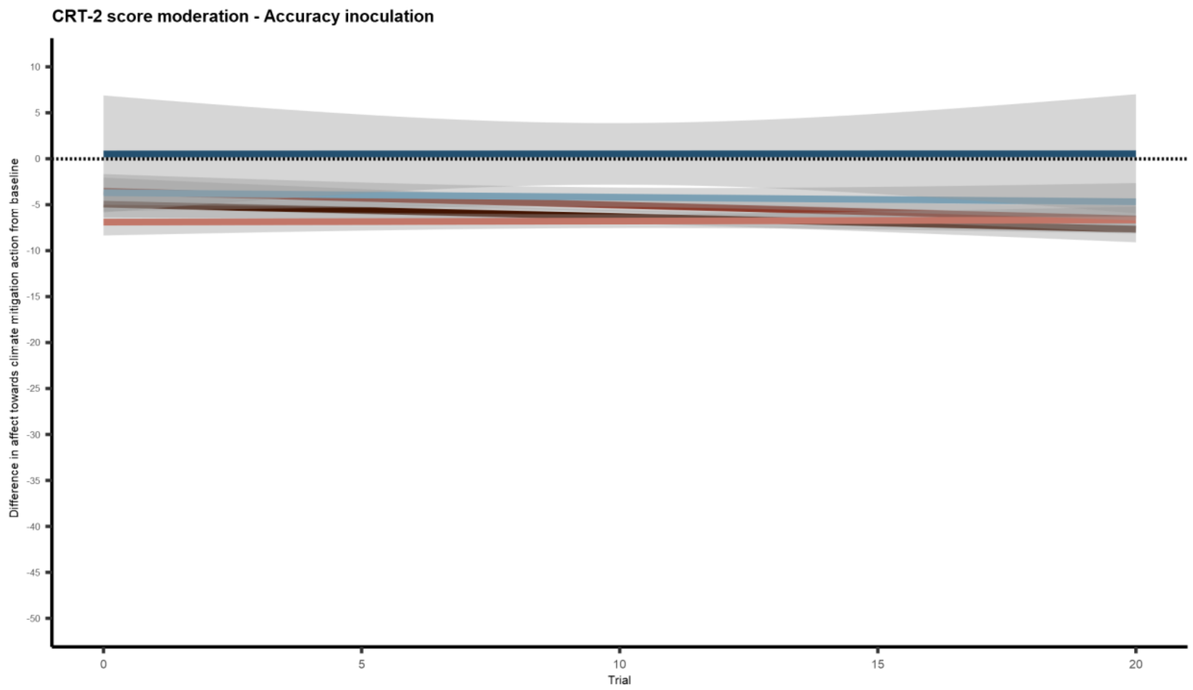

Supplementary Figure 13: Visual representation of the moderation by tendency for deliberate or intuitive thinking on the accuracy inoculation and mean affect towards climate mitigation action across the provision of twenty climate disinformation statements. Each color represents each level of tendency for deliberate thinking (CRT-2 score of 3 in represented light blue, CRT-2 score of 4 represented in dark blue) and intuitive thinking (CRT-2 score of 0 represented in dark red, CRT-2 score of 1 represented in red). The y axis represents the mean difference affect towards climate mitigation action from baseline (pre-inoculation and pre-disinformation provision), to better visualize the different slopes of CRT-2 scores, within condition and across trials. Values increasing from 0 are related to feeling overall more positively towards climate mitigation action, and values decreasing below 0 related to feeling overall more negatively towards climate mitigation action. The dashed line represents no mean difference from baseline. The x axis represents the trial number, with Trial=0 representing affect pre-intervention, and the numbers 1 to 20 representing each climate disinformation statement received. Light gray bands represent the mean-centered 95% CI produced by fitting a linear model. Color palette by MetBrewer package. Simple slope for CRT-2 scores: F_{ratio} (1, Inf)=2.859, p=.02. Simple slope of the two-way interaction between CRT-2 scores and trial: F_{ratio} (1, Inf)=5.316, p=.021.

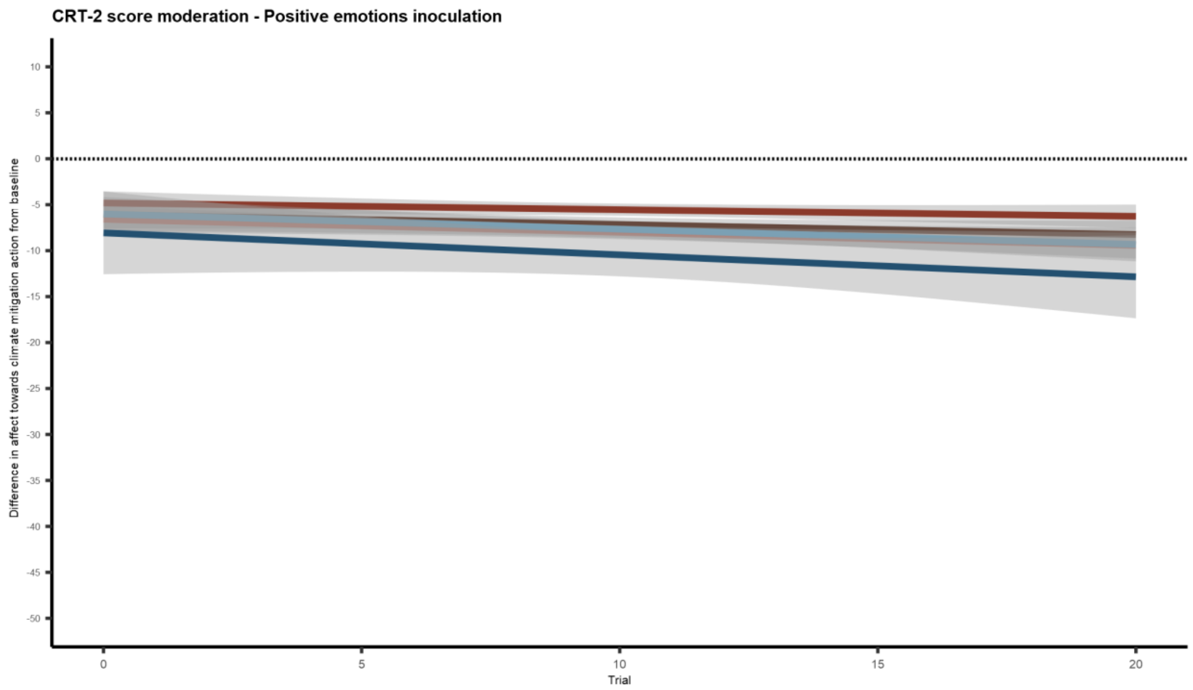

Supplementary Figure 14: Visual representation of the moderation by tendency for deliberate or intuitive thinking on the positive emotions inoculation and mean affect towards climate mitigation action across the provision of twenty climate disinformation statements. Each color represents each level of tendency for deliberate thinking (CRT-2 score of 3 in represented light blue, CRT-2 score of 4 represented in dark blue) and intuitive thinking (CRT-2 score of 0 represented in dark red, CRT-2 score of 1 represented in red). The y axis represents the mean difference affect towards climate mitigation action from baseline (pre-inoculation and pre-disinformation provision), to better visualize the different slopes of CRT-2 scores, within condition and across trials. Values increasing from 0 are related to feeling overall more positively towards climate mitigation action, and values decreasing below 0 related to feeling overall more negatively towards climate mitigation action. The dashed line represents no mean difference from baseline. The x axis represents the trial number, with Trial=0 representing affect pre-intervention, and the numbers 1 to 20 representing each climate disinformation statement received. Light gray bands represent the mean-centered 95% CI produced by fitting a linear model. Color palette by MetBrewer package. Simple slope for CRT-2 scores: F_{ratio} (1, Inf)=6.507, p=.011. Simple slope of the two-way interaction between CRT-2 scores and trial: F_{ratio} (1, Inf)=0.172, p=.68.

Supplementary Table 10 – Exploratory multilevel model for affect towards climate mitigation action across conditions when processing the twenty climate disinformation statement – moderation by political ideology.

Affect towards climate mitigation action						
				95% Confidence Intervals		
	Estimate	SE	t-value	Lower	Upper	P
Intercept	63.07	2.54	24.786	58.08	68.05	<.001
Age	-0.05	0.02	-2.635	-0.10	-0.01	.008
Gender	F-value(3, 1297.8):		5.6522			<.001
Political ideology	0.13	0.29	0.452	-0.43	0.69	.65
Trial	-0.17	0.05	-3.671	-0.27	-0.08	<.001
Condition	F-value(6, 6979):		2.3940			.025
Condition: Scientific consensus	3.38	2.58	1.312	-1.67	8.43	.19
Condition: Trust in scientists	4.65	2.53	1.822	-0.35	9.65	.07
Condition: Transparent communications	2.97	2.52	1.175	-1.98	7.92	.24
Condition: Moralization	7.83	2.52	3.103	2.88	12.77	.002
Condition: Accuracy	-2.98	2.55	-0.117	-5.29	4.69	.91
Condition: Positive emotions	3.17	2.59	1.223	-1.91	8.24	.22
Political ideology * Trial	-0.01	0.01	-0.871	-0.02	0.01	.38
Trial * Condition	F-value(6, 112994):		1.5633			.15
Trial * Condition: Scientific consensus	0.08	0.07	1.163	-0.05	0.21	.78
Trial * Condition: Trust in scientists	-0.01	0.07	-0.099	-0.14	0.13	.92
Trial * Condition: Transparent communications	-0.02	0.07	-0.321	-0.15	0.11	.75
Trial * Condition: Moralization	-0.09	0.07	-1.364	-0.22	0.04	.17
Trial * Condition: Accuracy	-0.06	0.07	-0.821	-0.19	0.08	.41
Trial * Condition: Positive emotions	0.06	0.07	0.903	-0.07	0.20	.37

Political ideology * Condition	F-value(6,6979):		1.6691			.12
Political ideology * Condition: Scientific consensus	-0.11	0.41	-0.278	-0.91	0.69	.78
Political ideology * Condition: Trust in scientists	-0.42	0.41	-1.018	-1.22	0.39	.31
Political ideology * Condition: Transparent communications	-0.35	0.41	-0.848	-1.15	0.45	.40
Political ideology * Condition: Moralization	-0.92	0.41	-2.259	-1.72	-0.12	.02
Political ideology * Condition: Accuracy	0.26	0.41	0.646	-0.54	1.06	.52
Political ideology * Condition: Positive emotions	-0.11	0.41	-0.275	-0.93	0.70	.78
Trial * Political ideology * Condition	F-value(6, 112994):		2.1846			.041
Trial * Political ideology * Condition: Scientific consensus	-0.21	0.11	-0.871	-0.04	0.0001	.051
Trial * Political ideology * Condition: Trust in scientists	-0.004	0.11	-0.353	-0.03	0.02	.72
Trial * Political ideology * Condition: Transparent communications	-0.01	0.11	-0.585	-0.03	0.01	.56
Trial * Political ideology * Condition: Moralization	0.02	0.11	1.454	-0.01	0.04	.15
Trial * Political ideology * Condition: Accuracy	-0.0003	0.11	-0.026	-0.02	0.02	.98
Trial * Political ideology * Condition: Positive emotions	-0.12	0.11	-1.122	-0.03	0.01	.26

Note: Condition contrast codes are in reference to the passive control condition. Two-sided tests, α corrected to .005 for condition contrasts.

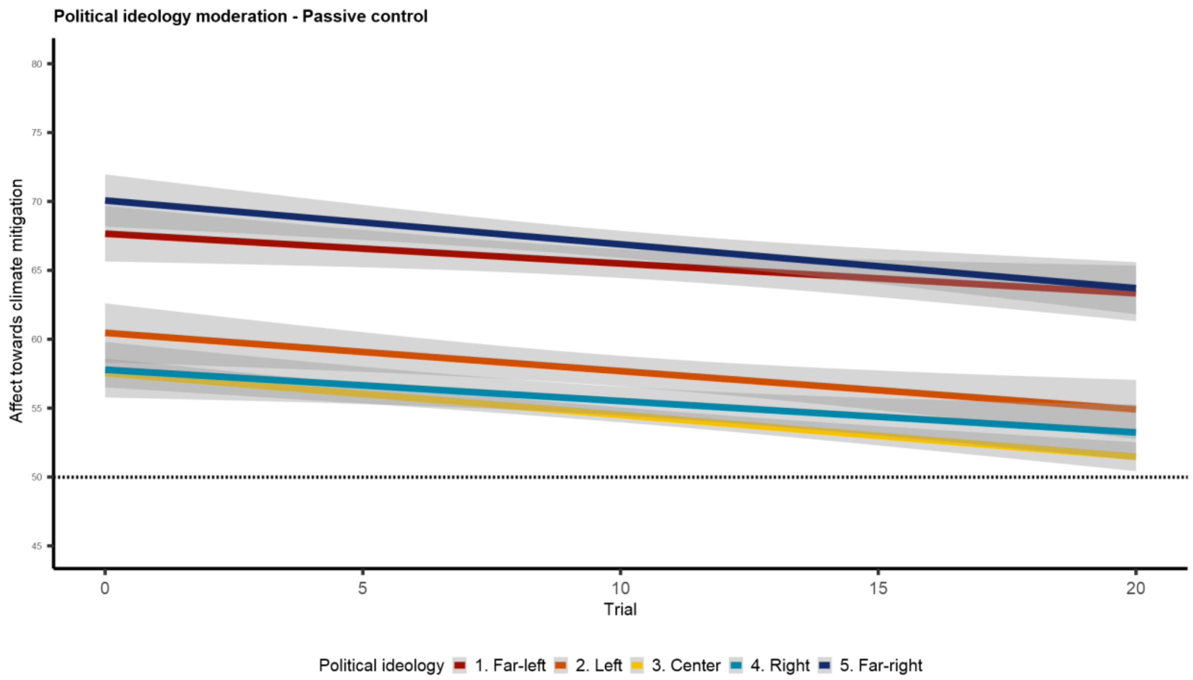

Supplementary Figure 15: Visual representation of the moderation by political ideology on the passive disinformation control condition and affect towards climate mitigation action across the provision of twenty climate disinformation statements. Each color represents a different interval of political ideology ratings (Far-left, in red: 1, 2; left, in orange: 3, 4; center, in yellow: 5, 6; right, in light blue: 7, 8; far-right, in blue: 9, 10). The y axis represents affect towards climate mitigation action, with values higher than 50 related to feeling increasingly more positively towards climate mitigation action, and values lower than 50 related to feeling more negatively towards climate mitigation action. Affect is represented in the interval between -45 and 80, to better visualize the simple slopes of political ideology within the condition. The dashed line represents no difference from baseline. The x axis represents the trial number, with Trial=0 representing affect pre-intervention, and the numbers 1 to 20 representing each climate disinformation statement received. Light gray bands represent the 95% CI produced by fitting a linear model. Color palette by MetBrewer package. Simple slope for political ideology: F_{ratio} (1, Inf)=0.048, p=.83. Simple slope of the two-way interaction between political ideology and trial: F_{ratio} (1, Inf)=0.758, p=.38.

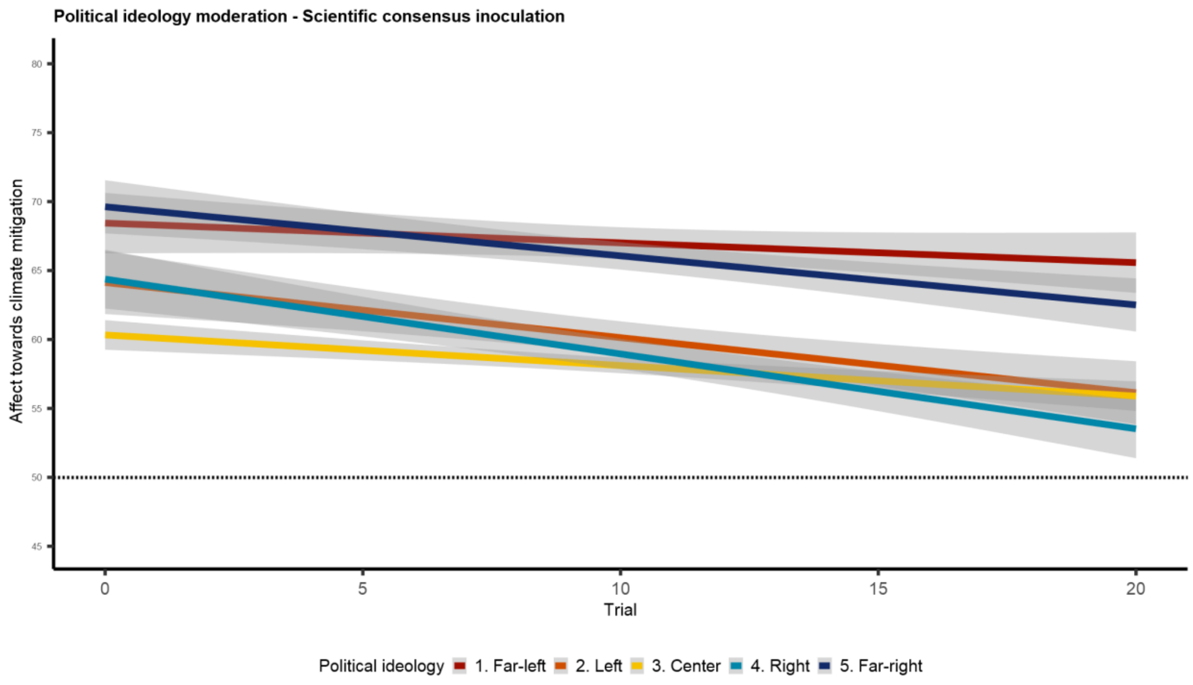

Supplementary Figure 16: Visual representation of the moderation by political ideology on the scientific consensus inoculation and mean affect towards climate mitigation action across the provision of twenty climate disinformation statements. Each color represents a different interval of political ideology ratings (Far-left, in red: 1, 2; left, in orange: 3, 4; center, in yellow: 5, 6; right, in light blue: 7, 8; far-right, in blue: 9, 10). The y axis represents mean affect towards climate mitigation action, with values higher than 50 related to feeling increasingly more positively towards climate mitigation action, and values lower than 50 related to feeling more negatively towards climate mitigation action. Affect is represented in the interval between -45 and 80, to better visualize the simple slopes of political ideology within the condition. The dashed line represents no mean difference from baseline. The x axis represents the trial number, with Trial=0 representing affect pre-intervention, and the numbers 1 to 20 representing each climate disinformation statement received. Light gray bands represent the mean-centered 95% CI produced by fitting a linear model. Color palette by MetBrewer package. Simple slope for political ideology: F_{ratio} (1, Inf)=0.960, p=.33. Simple slope of the two-way interaction between political ideology and trial: F_{ratio} (1, Inf)=12.807, p=.003.

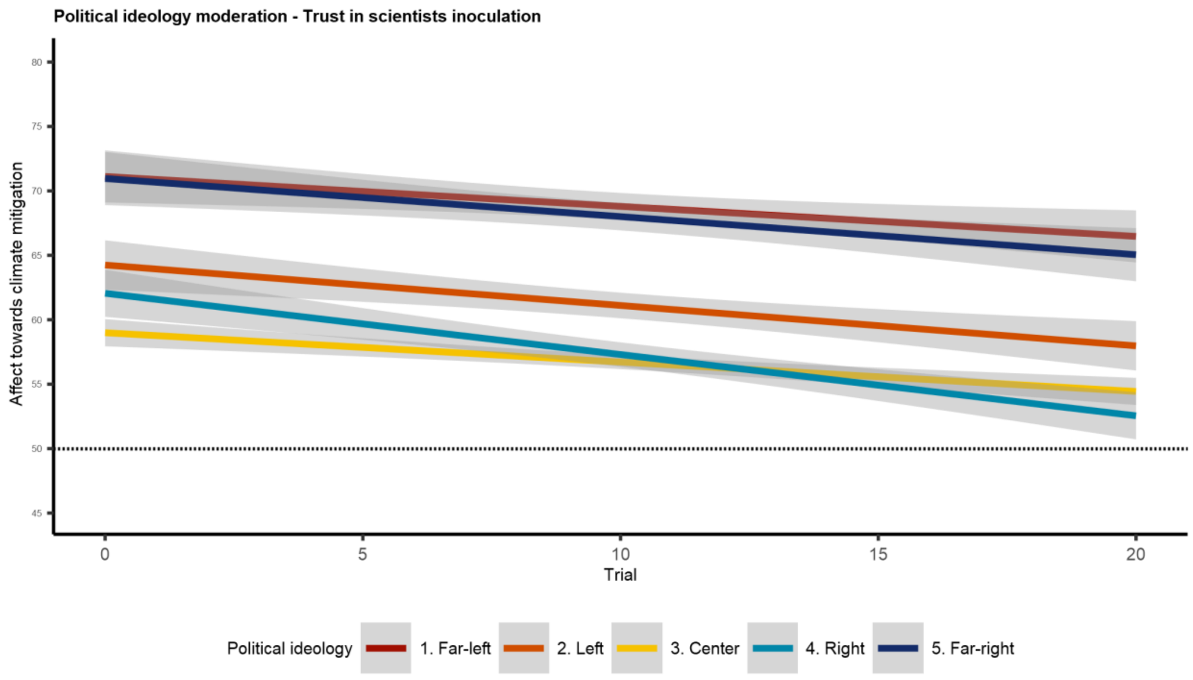

Supplementary Figure 17: Visual representation of the moderation by political ideology on the trust in scientists inoculation and mean affect towards climate mitigation action across the provision of twenty climate disinformation statements. Each color represents a different interval of political ideology ratings (Far-left, in red: 1, 2; left, in orange: 3, 4; center, in yellow: 5, 6; right, in light blue: 7, 8; far-right, in blue: 9, 10). The y axis represents mean affect towards climate mitigation action, with values higher than 50 related to feeling increasingly more positively towards climate mitigation action, and values lower than 50 related to feeling more negatively towards climate mitigation action. Affect is represented in the interval between -45 and 80, to better visualize the simple slopes of political ideology within the condition. The dashed line represents no mean difference from baseline. The x axis represents the trial number, with Trial=0 representing affect pre-intervention, and the numbers 1 to 20 representing each climate disinformation statement received. Light gray bands represent the mean-centered 95% CI produced by fitting a linear model. Color palette by MetBrewer package. Simple slope for political ideology: F_{ratio} (1, Inf)=1.977, p=.16. Simple slope of the two-way interaction between political ideology and trial: F_{ratio} (1, Inf)=1.794, p=.18.

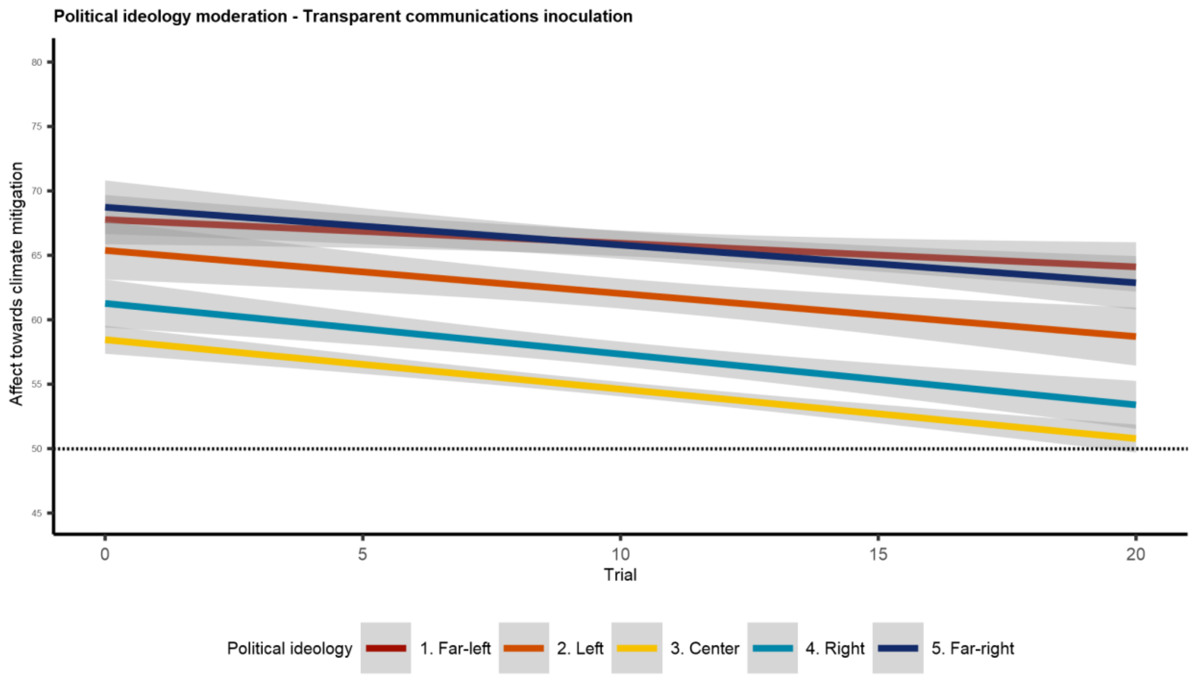

Supplementary Figure 18: Visual representation of the moderation by political ideology on the transparent communications inoculation and mean affect towards climate mitigation action across the provision of twenty climate disinformation statements. Each color represents a different interval of political ideology ratings (Far-left, in red: 1, 2; left, in orange: 3, 4; center, in yellow: 5, 6; right, in light blue: 7, 8; far-right, in blue: 9, 10). The y axis represents mean affect towards climate mitigation action, with values higher than 50 related to feeling increasingly more positively towards climate mitigation action, and values lower than 50 related to feeling more negatively towards climate mitigation action. Affect is represented in the interval between -45 and 80, to better visualize the simple slopes of political ideology within the condition. The dashed line represents no mean difference from baseline. The x axis represents the trial number, with Trial=0 representing affect pre-intervention, and the numbers 1 to 20 representing each climate disinformation statement received. Light gray bands represent the mean-centered 95% CI produced by fitting a linear model. Color palette by MetBrewer package. Simple slope for political ideology: F_{ratio} (1, Inf)=1.584, p=.21. Simple slope of the two-way interaction between political ideology and trial: F_{ratio} (1, Inf)=2.798, p=.09.

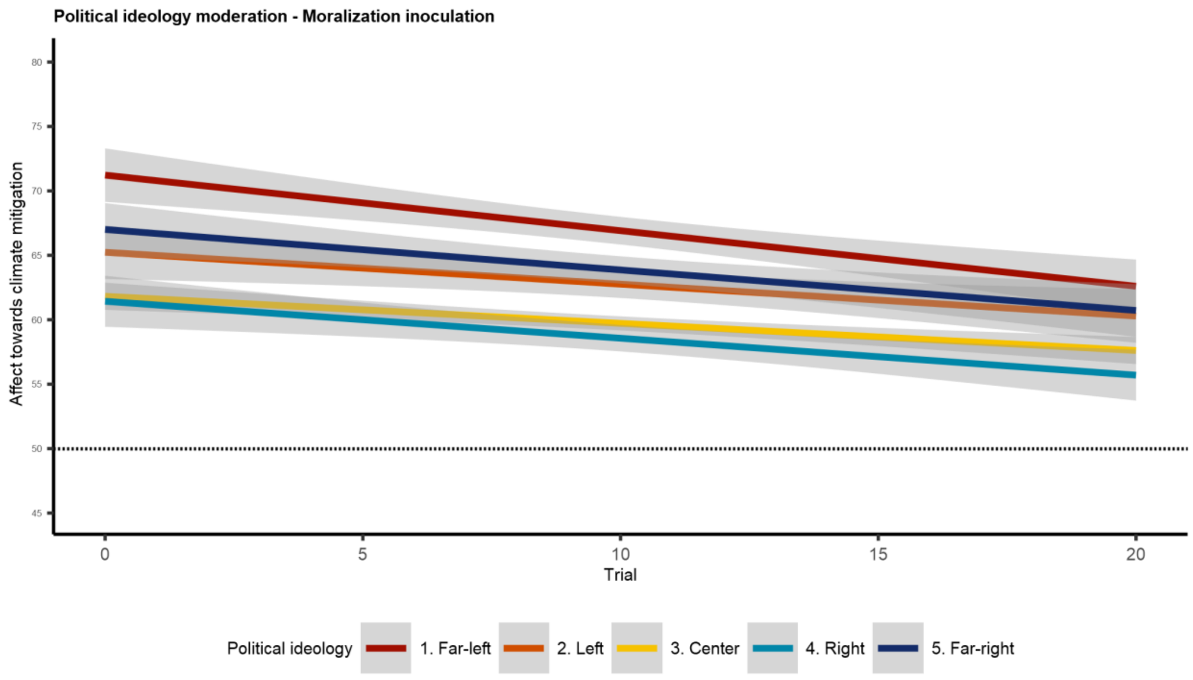

Supplementary Figure 19: Visual representation of the moderation by political ideology on the moralization inoculation and mean affect towards climate mitigation action across the provision of twenty climate disinformation statements. Each color represents a different interval of political ideology ratings (Far-left, in red: 1, 2; left, in orange: 3, 4; center, in yellow: 5, 6; right, in light blue: 7, 8; far-right, in blue: 9, 10). The y axis represents mean affect towards climate mitigation action, with values higher than 50 related to feeling increasingly more positively towards climate mitigation action, and values lower than 50 related to feeling more negatively towards climate mitigation action. Affect is represented in the interval between -45 and 80, to better visualize the simple slopes of political ideology within the condition. The dashed line represents no mean difference from baseline. The x axis represents the trial number, with Trial=0 representing affect pre-intervention, and the numbers 1 to 20 representing each climate disinformation statement received. Light gray bands represent the mean-centered 95% CI produced by fitting a linear model. Color palette by MetBrewer package. Simple slope for political ideology: F_{ratio} (1, Inf)=6.182, p=.013. Simple slope of the two-way interaction between political ideology and trial: F_{ratio} (1, Inf)=1.399, p=.24.

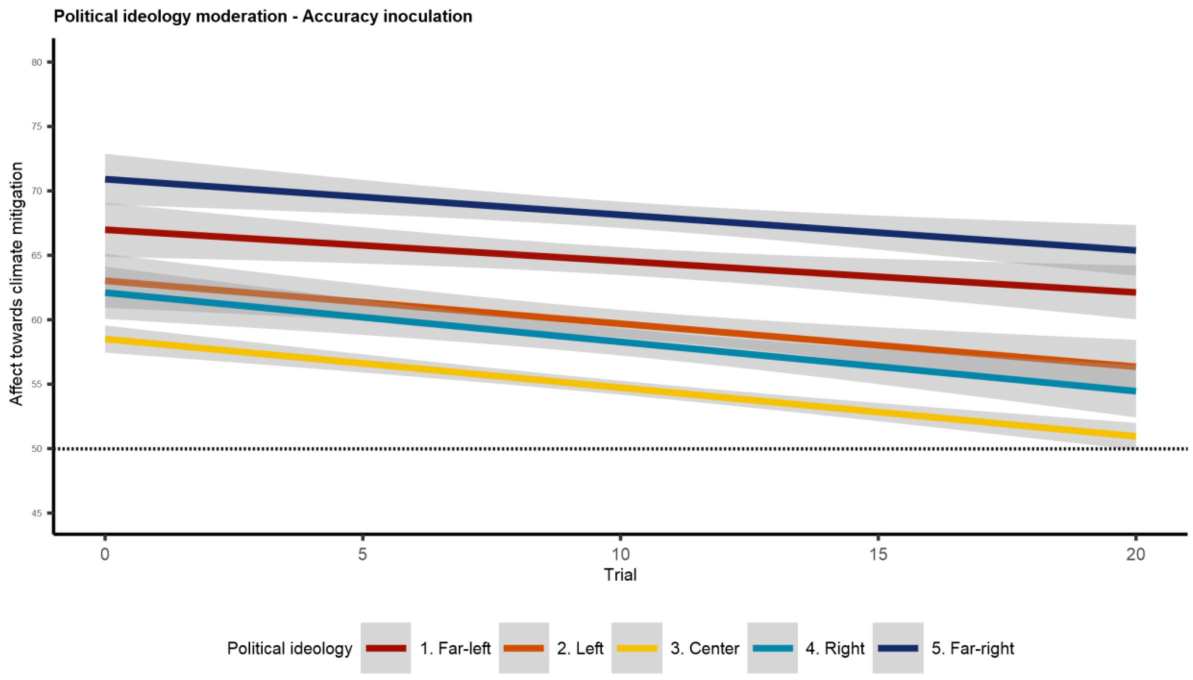

Supplementary Figure 20: Visual representation of the moderation by political ideology on the accuracy inoculation and mean affect towards climate mitigation action across the provision of twenty climate disinformation statements. Each color represents a different interval of political ideology ratings (Far-left, in red: 1, 2; left, in orange: 3, 4; center, in yellow: 5, 6; right, in light blue: 7, 8; far-right, in blue: 9, 10). The y axis represents mean affect towards climate mitigation action, with values higher than 50 related to feeling increasingly more positively towards climate mitigation action, and values lower than 50 related to feeling more negatively towards climate mitigation action. Affect is represented in the interval between -45 and 80, to better visualize the simple slopes of political ideology within the condition. The dashed line represents no mean difference from baseline. The x axis represents the trial number, with Trial=0 representing affect pre-intervention, and the numbers 1 to 20 representing each climate disinformation statement received. Light gray bands represent the mean-centered 95% CI produced by fitting a linear model. Color palette by MetBrewer package. Simple slope for political ideology: F_{ratio} (1, Inf)=1.310, p=.25. Simple slope of the two-way interaction between political ideology and trial: F_{ratio} (1, Inf)=0.790, p=.37.

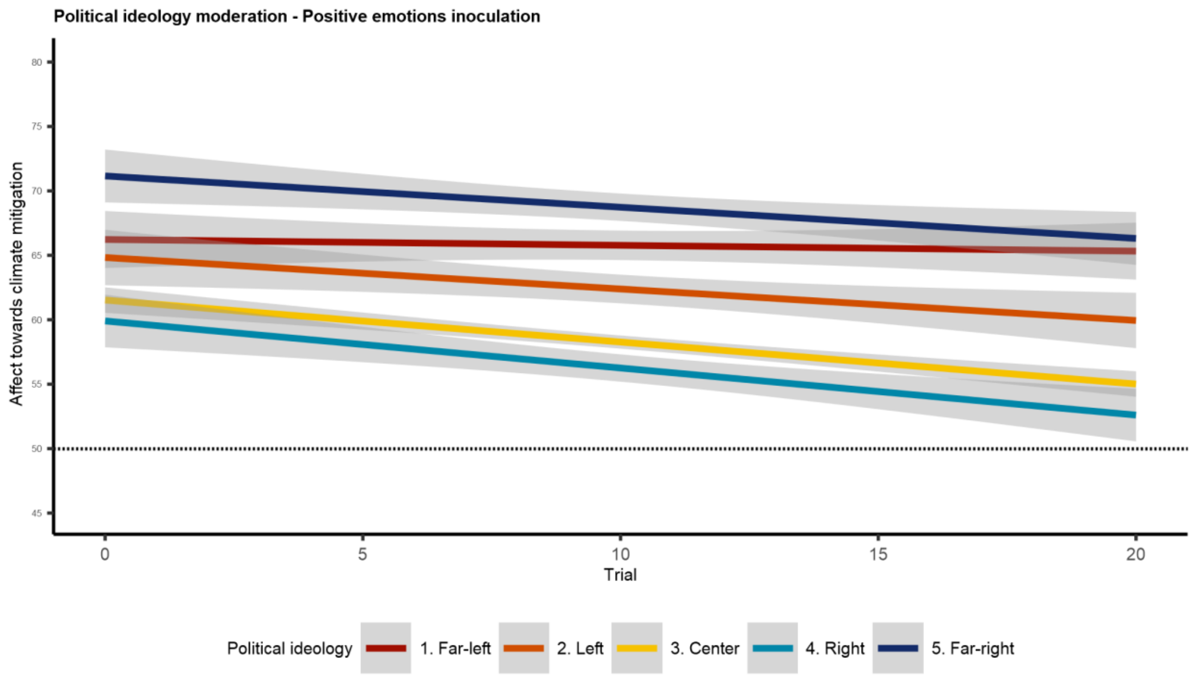

Supplementary Figure 21: Visual representation of the moderation by political ideology on the positive emotions inoculation and mean affect towards climate mitigation action across the provision of twenty climate disinformation statements. Each color represents a different interval of political ideology ratings (Far-left, in red: 1, 2; left, in orange: 3, 4; center, in yellow: 5, 6; right, in light blue: 7, 8; far-right, in blue: 9, 10). The y axis represents mean affect towards climate mitigation action, with values higher than 50 related to feeling increasingly more positively towards climate mitigation action, and values lower than 50 related to feeling more negatively towards climate mitigation action. Affect is represented in the interval between -45 and 80, to better visualize the simple slopes of political ideology within the condition. The dashed line represents no mean difference from baseline. The x axis represents the trial number, with Trial=0 representing affect pre-intervention, and the numbers 1 to 20 representing each climate disinformation statement received. Light gray bands represent the mean-centered 95% CI produced by fitting a linear model. Color palette by MetBrewer package. Simple slope for political ideology: F_{ratio} (1, Inf)=0.405, p=.52. Simple slope of the two-way interaction between political ideology and trial: F_{ratio} (1, Inf)=5.623, p=.018.

Supplementary Table 11 – Exploratory multilevel models for participants' belief in the reality, anthropogenic causes, and negativity of the consequences of climate change – moderation by political ideology.

Belief in the reality of climate change

Predictor	Estimate	SE	t-value	95% Confidence Intervals: Lower	95% Confidence Intervals: Upper	P
Intercept	4.24	0.10	40.710	4.04	4.44	$<.2^{e-16}$
Age	0.001	0.001	0.759	-0.001	0.003	.45
Gender	F-value(3, 5939.5):		3.1303			.024
Political ideology	-0.50	0.01	-3.921	-0.07	-0.02	$.9^{e-05}$
Condition	F-value(6, 5936.9):			0.9304		.47
Condition: Scientific consensus	0.08	0.11	0.715	-0.14	0.30	.47
Condition: Trust in scientists	0.04	0.11	0.386	-0.18	0.26	.70
Condition: Transparent commu-nications	-0.02	0.11	-0.179	-0.24	0.20	.86
Condition: Morali-zation	0.11	0.11	1.012	-0.11	0.33	.31
Condition: Accuracy	0.07	0.11	0.580	-0.16	0.29	.56

Belief in the anthropogenic causes of climate change

Predictor	Estimate	SE	t-value	95% Confidence Intervals: Lower	95% Confidence Intervals: Upper	p
Intercept	4.26	0.11	38.470	4.04	4.48	$<.2^{e-16}$
Age	-0.003	0.001	-2.860	-0.004	-0.001	.004
Gender	F-value(3, 5938.8):		4.8094			.002
Political ideology	-0.04	0.01		-0.07	-0.01	.002
Condition	F-value(6, 5936.7):		0.2014			.98
Condition: Scientific consensus	-0.06	0.12	-0.544	-0.29	0.16	.59
Condition: Trust in scientists	-0.003	0.12	-0.025	-0.22	0.22	.98
Condition: Transparent commu-nications	-0.05	0.12	-0.408	-0.27	0.18	.68
Condition: Morali-zation	0.01	0.12	0.059	-0.22	0.23	.95
Condition: Accuracy	-0.09	0.12	-0.789	-0.32	0.13	.43

Belief in the negativity of the consequences of climate change

Predictor	Estimate	SE	t-value	95% Confidence Intervals: Lower	95% Confidence Intervals: Upper	p
Intercept	4.23	0.10	42.415	4.04	4.43	$<.2^{e-16}$
Age	-0.001	0.001	-1.004	-0.002	0.001	.32
Gender	F-value(3, 5939.4):		5.7349			.0006
Political ideology	-0.04	0.01	-3.446	-0.07	-0.02	.0006
Condition	F-value(6, 5936.9):			0.8321		.54
Condition: Scientific consensus	-0.09	0.11	-0.818	-0.30	0.12	.25
Condition: Trust in scientists	-0.004	0.11	-0.042	-0.22	0.21	.32
Condition: Transparent commu-nications	-0.10	0.11	-0.941	-0.31	0.11	.41
Condition: Morali-zation	0.01	0.11	0.113	-0.20	0.22	.97
Condition: Accuracy	-0.10	0.11	-0.920	-0.31	0.11	.36

Condition: Positive emotions	0.21	0.11	1.874	-0.10	0.44	.06	-0.03	0.12	-0.237	-0.26	0.20	.81	0.09	0.11	0.784	-0.13	0.30	.43
Political ideology * Condition	F-value(6, 5937.1):		0.6733			.67	F-value(6, 5937.1):	0.2317				.97	F-value(6, 5937.0):	0.9014				.49
Political ideology * Condition: Scientific consensus	-0.001	0.02	-0.058	-0.04	0.03	.95	0.003	0.02	0.181	-0.03	0.04	.86	0.01	0.02	0.807	-0.02	0.05	.42
Political ideology * Condition: Trust in scientists	-0.004	0.02	-0.234	-0.04	0.03	.82	-0.001	0.02	0.043	-0.04	0.04	.97	-0.002	0.02	-0.106	-0.04	0.03	.92
Political ideology * Condition: Transparent commu-nications	0.01	0.02	0.778	-0.02	0.05	.44	0.10	0.02	0.555	-0.03	0.05	.58	0.02	0.02	0.937	-0.02	0.05	.35
Political ideology * Condition: Morali-zation	-0.003	0.02	-0.154	-0.04	003	.88	-0.001	0.02	-0.032	-0.04	0.04	.97	0.004	0.02	0.237	-0.03	0.04	.81
Political ideology * Condition: Accuracy	-0.003	0.02	-0.186	-0.04	0.03	.85	0.02	0.02	0.866	-0.04	0.05	.39	0.02	0.02	0.966	-0.02	0.05	.33
Political ideology * Condition: Positive emotions	-0.02	0.02	-1.243	-0.06	0.01	.21	0.001	0.02	0.027	-0.04	0.04	.97	-0.02	0.02	-0.907	-0.05	0.02	.36

Note: Condition contrast codes are in reference to the passive control condition. Two-sided tests, α corrected to .005 for condition contrasts.

Supplementary Table 12 – Exploratory multilevel model for WEPT performance and truth discrimination score – moderation by political ideology.

WEPT Performance (Poisson, zero-inflated)	Truth discernment score
95% Confidence Intervals	95% Confidence Intervals

	Estimate	SE	t-value	Lower	Upper	p	Estimate	SE	t-value	Lower	Upper	p
Intercept	0.45	0.09	4.838	0.27	0.64	$.1^{e-06}$	1.12	0.22	51.284	10.74	11.59	$<.2^{e-16}$
Age	0.01	0.001	16.416	0.01	0.02	$<.2^{e-16}$	0.03	0.002	12.482	0.02	0.03	$<.2^{e-16}$
Gender	$\chi(3)$:		13.4409			.004	F-value(3, 5941.7):		2.4585			.06
Political ideology	0.003	0.01	-0.224	-0.02	0.02	.82	-0.13	0.03	-4.649	-0.19	-0.08	$.3^{e-06}$
Condition	$\chi(6)$:		11.8472			.07	F-value(3, 5941.7):		0.6961			.65
Condition: Scientific consensus	0.04	0.10	0.353	-0.17	0.24	.72	-0.17	0.26	-0.656	-0.67	0.33	.51
Condition: Trust in scientists	-0.03	0.11	-0.259	-0.24	0.18	.80	0.05	0.25	0.210	-0.44	0.55	.83
Condition: Transparent communications	0.03	0.10	0.307	-0.17	0.23	.76	-0.21	0.25	-0.823	-0.69	0.28	.41
Condition: Moralization	0.04	0.11	0.387	-0.17	0.25	.70	-0.19	0.25	-0.781	-0.68	0.29	.44
Condition: Accuracy	-0.003	0.11	-0.027	-0.22	0.21	.98	0.17	0.25	0.662	-0.33	0.66	.51
Condition: Positive emotions	0.12	0.10	1.130	-0.09	0.32	.26	-0.19	0.26	-0.744	-0.69	0.31	.46
Political ideology * Condition	$\chi(6)$:		2.0480			.92	F-value(3, 5937.9):		0.5822			.74
Political ideology * Condition: Scientific consensus	-0.004	0.02	-0.224	-0.04	0.03	.82	0.04	0.04	0.914	-0.04	0.12	.36
Political ideology * Condition: Trust in scientists	-0.01	0.02	-0.735	-0.05	0.03	.46	0.02	0.04	0.604	-0.06	0.10	.55
Political ideology * Condition: Transparent communications	-0.01	0.02	-0.735	-0.05	0.02	.46	0.07	0.04	1.648	-0.01	0.15	.10
Political ideology * Condition: Moralization	-0.02	0.02	-0.887	-0.05	0.02	.38	0.05	0.04	1.260	-0.03	0.13	.21
Political ideology * Condition: Accuracy	-0.01	0.02	-0.673	-0.05	.002	.50	0.03	0.04	0.829	-0.05	0.11	.41
Political ideology *	-0.02	0.02	-1.215	-0.05	0.01	.22	0.05	0.04	1.298	--0.03	0.13	.19

Condition: Positive emotions						
Zero-inflated intercept	-0.12	0.03	-4.021	-0.17	-0.06	<.2^{e-16}

Note: Condition contrast codes are in reference to the passive control condition. Two-sided tests, α corrected to .005 for condition contrasts.

Supplementary Table 13 – Preregistered multilevel model for affect towards climate mitigation action across conditions when processing the twenty climate disinformation statement – moderation by demand effects on psychological inoculations.

Affect towards climate mitigation action						
				95% Confidence Intervals		
	Estimate	SE	t-value	Lower	Upper	P
Intercept	64.67	2.07	31.252	60.61	68.72	<.2^{e-16}
Age	-0.06	0.02	-2.832	-0.01	-0.02	.008
Gender	F-value(3, 5936):		5.5356			.0009
Political ideology	-0.21	0.11	-1.914	-0.42	0.005	.06
Trial	-0.21	0.02	-10.559	-0.25	-0.17	<.2^{e-16}
Condition	F-value(6, 6926):		2.1923			.041
Condition: Scientific consensus	3.02	1.10	2.748	0.87	5.17	.006
Condition: Trust in scientists	2.67	1.09	2.449	0.53	4.80	.014
Condition: Transparent communications	1.44	1.09	1.319	-0.70	3.58	.19
Condition: Moralization	2.65	1.08	1.727	0.83	5.08	.006
Condition: Accuracy	1.45	1.11	1.319	-0.72	3.61	.19
Condition: Positive emotions	2.93	1.09	2.684	0.79	5.07	.007
Trial * Condition	F-value(6, 112955):		1.4749			.18
Trial * Condition: Scientific consensus	-0.05	0.03	-1.493	-0.10	0.01	.14
Trial * Condition: Trust in scientists	-0.03	0.03	-0.984	-0.08	0.03	.32
Trial * Condition: Transparent communications	-0.06	0.03	-1.943	-0.11	0.005	.052
Trial * Condition: Moralization	-0.08	0.03	-0.114	-0.06	0.05	.91
Trial * Condition: Accuracy	-0.06	0.03	-1.984	-0.11	-0.001	.047

Trial * Condition: Positive emotions	-0.08	0.03	-0.820	-0.06	0.05	.78
Demand effect * Condition	F-value(6,5935):		1.2125			.30
Demand effect * Condition: Scientific consensus	-3.83	2.48	-1.540	-8.69	1.04	.12
Demand effect * Condition: Trust in scientists	-4.49	2.61	-1.722	-9.59	0.62	.09
Demand effect * Condition: Transparent communications	-5.76	2.52	-2.301	-10.72	-0.85	.021
Demand effect * Condition: Moralization	-4.34	2.34	-1.851	-8.93	0.26	.06
Demand effect * Condition: Accuracy	-5.12	2.18	-2.347	-9.39	-0.84	.019
Demand effect * Condition: Positive emotions	-5.57	2.48	-2.247	-10.42	-0.71	.025

Note: Condition contrast codes are in reference to the passive control condition. Two-sided tests, α corrected to .005 for condition contrasts.

Supplementary Table 14 – Preregistered multilevel model for affect towards climate mitigation action across conditions when processing the twenty climate disinformation statement – moderation by demand effects.

Affect towards climate mitigation action (Passive control condition)						
				95% Confidence Intervals		
	Estimate	SE	t-value	Lower	Upper	P
Intercept	61.65	3.16	19.491	55.45	67.85	<.2^{e-16}
Age	-0.06	0.01	-1.107	-0.17	0.05	.27
Gender	F-value(3,846.6):		0.1806			.91
Demand effect	6.199	1.96	3.167	2.36	10.03	.002
Trial	-0.17	0.05	-3.671	-0.25	-0.17	<.2^{e-16}
Trial * Demand effect	-0.02	0.05	-0.386	-0.13	0.08	.70

Note: Condition contrast codes are in reference to the passive control condition. Two-sided tests, α=.05.
