# Supplementary material for: Psychological inoculation strategies to fight climate disinformation across 12 countries
Source: Nat Hum Behav. 2023 Nov 30;8(2):380–98. doi: 10.1038/s41562-023-01736-0 (PMC10896732; doi:10.1038/s41562-023-01736-0)
Supplement: Supplementary file 2 — Reporting Summary [file 41562_2023_1736_MOESM2_ESM.pdf]

nature portfolio

Corresponding author(s): Tobia Spampatti

Last updated by author(s): 2023/08/21

# Reporting Summary

Nature Portfolio wishes to improve the reproducibility of the work that we publish. This form provides structure for consistency and transparency in reporting. For further information on Nature Portfolio policies, see our Editorial Policies and the Editorial Policy Checklist.

## Statistics

For all statistical analyses, confirm that the following items are present in the figure legend, table legend, main text, or Methods section.

| n/a | Confirmed | |
|---|---|---|
| ☐ | ☒ | The exact sample size (*n*) for each experimental group/condition, given as a discrete number and unit of measurement |
| ☐ | ☒ | A statement on whether measurements were taken from distinct samples or whether the same sample was measured repeatedly |
| ☐ | ☒ | The statistical test(s) used AND whether they are one- or two-sided<br>*Only common tests should be described solely by name; describe more complex techniques in the Methods section.* |
| ☐ | ☒ | A description of all covariates tested |
| ☐ | ☒ | A description of any assumptions or corrections, such as tests of normality and adjustment for multiple comparisons |
| ☐ | ☒ | A full description of the statistical parameters including central tendency (e.g. means) or other basic estimates (e.g. regression coefficient) AND variation (e.g. standard deviation) or associated estimates of uncertainty (e.g. confidence intervals) |
| ☐ | ☒ | For null hypothesis testing, the test statistic (e.g. $F$, $t$, $r$) with confidence intervals, effect sizes, degrees of freedom and $P$ value noted<br>*Give P values as exact values whenever suitable.* |
| ☐ | ☒ | For Bayesian analysis, information on the choice of priors and Markov chain Monte Carlo settings |
| ☐ | ☒ | For hierarchical and complex designs, identification of the appropriate level for tests and full reporting of outcomes |
| ☐ | ☒ | Estimates of effect sizes (e.g. Cohen's *d*, Pearson's *r*), indicating how they were calculated |

*Our web collection on statistics for biologists contains articles on many of the points above.*

## Software and code

Policy information about availability of computer code

| | |
|---|---|
| Data collection | We used the online Qualtrics XM Platform (2020 version) for data collection, accessed through institutional license dispensed to the corresponding author. |
| Data analysis | We used  open software R (Version 4.1.3, 2023.03.0+386). Within the R environment, we used packages "cowplot" (Version 1.1.1), "ggdist" (Version 3.1.1), "lmerTest" (Version 3.1.3), "lme4" (Version 1.1-27.1), "TOSTER" (Version 0.3.4), "MuMIn" (Version 1.46.0), "sjmisc" (Version 2.8.9), "sjPlot" (Version 2.8.10), "effects" (Version 4.2.1), "car" (Version 3.1.1), "pscl" (Version 1.5.5), "DescTools" (Version 0.99.27), "forcats" (Version 0.5.1), "stringr" (Version 1.4.0), "dyplr" (Version 1.0.10), "purr" (Version 0.3.4), "readr" (Version 2.1.2), "tidyr" (Version 1.2.0), "tibble" (Version 3.1.6), "tidyverse" (Version 1.3.1), "gmodels" (Version 2.18.1), "psych" (Version 2.2.3), "mlogit" (Version 1.1.1), "Hmisc" (Version 4.6.0), "QuantPsych" (Version 1.5), "MASS" (Version 7.3.56), "boot" (Version 1.3.28), "sandwich" (Version 3.0.1), "cocor" (Version 1.1.3), "MSwM" (Version 1.5), "emmeans" (Version 1.8.2), "Rmisc" (Version 1.5), "plyr" (Version 1.8.8), "lattice" (Version 0.20.45), "raincloudplots" (Version 0.2.0), "ggplot" (Version 2_3.3.5), "MetBrewer" (Version 0.2.0), "r2glmm" (Version 0.1.2), "visreg" (Version 2.7.0), "glmTMB" (Version 1.1.7), "DHARMa" (Version 0.4.5). |

For manuscripts utilizing custom algorithms or software that are central to the research but not yet described in published literature, software must be made available to editors and reviewers. We strongly encourage code deposition in a community repository (e.g. GitHub). See the Nature Portfolio guidelines for submitting code & software for further information.

# Data

Policy information about availability of data

All manuscripts must include a data availability statement. This statement should provide the following information, where applicable:

- Accession codes, unique identifiers, or web links for publicly available datasets
- A description of any restrictions on data availability
- For clinical datasets or third party data, please ensure that the statement adheres to our policy

Anonymized data, Qualtrics files and stimuli are available on the OSF at https://osf.io/m58zx/?view_only=95fd430f4b7e4ee99c9c8b472e31d6b3.

# Research involving human participants, their data, or biological material

Policy information about studies with human participants or human data. See also policy information about sex, gender (identity/presentation), and sexual orientation and race, ethnicity and racism.

| | |
|---|---|
| Reporting on sex and gender | Only self-reported gender identity was collected (1-item: "Which gender do you identify with?" Responses: "Male"; "Female"; "non-binary / third gender"; "prefer not to answer". As per our preregistration, no participant was excluded from their response to this question. No sex data was collected. Gender self-report was utilized as a covariate in all multilevel models reported in the manuscript. |
| Reporting on race, ethnicity, or other socially relevant groupings | We did not collect categorization data for socially relevant groupings. |
| Population characteristics | Mean participant age = 39.15 ± 14.17, n=3555 female. Participants were quota-sampled by age and gender within twelve (12) countries: USA, Canada, UK, Ireland, Australia, New Zealand, Singapore, Philippines, India, Pakistan, Nigeria, and South Africa |
| Recruitment | Participants were recruited for the online survey by the panel provider Market Science Institute (https://site.msi-aci.com/). A potential selection bias that might impact the generizability of the results is that participation to the survey was conditional to having a device (laptop, tablet, or phone) with an internet connection. |
| Ethics oversight | The study was approved by the Ethics Committee of the University of Geneva (https://cureg.unige.ch/en/). |

Note that full information on the approval of the study protocol must also be provided in the manuscript.

# Field-specific reporting

Please select the one below that is the best fit for your research. If you are not sure, read the appropriate sections before making your selection.

☐ Life sciences ☒ Behavioural & social sciences ☐ Ecological, evolutionary & environmental sciences

For a reference copy of the document with all sections, see nature.com/documents/nr-reporting-summary-flat.pdf

# Behavioural & social sciences study design

All studies must disclose on these points even when the disclosure is negative.

| | |
|---|---|
| Study description | Quantitative experimental study. Mixed design. Participants were randomly assigned to one of eight different between-participants conditions: pure control (no inoculation, no disinformation), passive disinformation control (disinformation without inoculation), scientific consensus inoculation, trust in scientists inoculation, transparent communication inoculation, moralization of climate action inoculation, accuracy inoculation, and positive emotion inoculation. We chose a passive disinformation control condition over an active or positive control in order to better mimic real-life information environments, where climate disinformation is most frequently encountered passively and in multiple occurrences. Participants and experimenters were blind to the name and aim of the condition that participants were randomized into (double blind). The experiment contained twenty within-participants repeated measures of affect towards climate mitigation actions, assessed after each of the twenty climate disinformation statements. |
| Research sample | We collected one quota-based sample (age and gender) from each of twelve (12) countries: USA, Canada, UK, Ireland, Australia, New Zealand, Singapore, Philippines, India, Pakistan, Nigeria, and South Africa.<br>We collected the sample with quota for gender and age from the panel provider Market Science Institute. The sample comprised of participants from twelve countries, n = 568 participants per country, for a total of N = 6816 (mean age = 39.15 ± 14.17, n=3555 female) participants.<br>We choose these countries for two reasons. First, as all the real and validated climate disinformation material (see Table 3 in the manuscript) was in the English language, we selected these countries as they all list English as one of their official languages. Second, we selected these countries to have a broad representativeness across continents and beyond WEIRD samples. |
| Sampling strategy | We collected one quota-based sample (age and gender) from each of twelve (12) countries: USA, Canada, UK, Ireland, Australia, New |

| | |
|---|---|
| Sampling strategy | Zealand, Singapore, Philippines, India, Pakistan, Nigeria, and South Africa.<br>We collected the sample with quota for gender and age from the panel provider Market Science Institute. The sample comprised of participants from twelve countries, n = 568 participants per country, for a total of N = 6816 (mean age = 39.15 ± 14.17, n=3555 female) participants. We identified the required sample size a-priori, with G*Power (Version 3.0), in order to have 95% power to detect a difference between any intervention condition and the passive disinformation control condition of $\delta = 0.20$ in a one-tailed t-test with $\alpha = .005$, for all main hypotheses separately. We selected the smallest effect size of interest (SESOI) from the lower bound of the confidence interval of the meta-analytically identified effect size of fact-checking interventions on political topics, as we reasoned that a new disinformation intervention would be of interest if and only if it has an effect that is larger than already available interventions such as fact-checks. Incidentally, a recent paper showed that the effects of more established psychological inoculations on sharing intentions of manipulative content is $\delta = 0.20$, increasing our confidence in the practical interest of this SESOI. |
| Data collection | Participation was entirely online, the survey being accessed through an anonymous link.<br>Participants and experimenters were blind to the name and aim of the condition that participants were randomized into (double blind).<br>Procedure: Participants accessed the survey through an anonymous link made available by the panel provider, and provided their explicit consent to the study. After consenting, participants reported their demographics (gender, age, education, and political orientation: single-item, 10-point scale: 1=[Extreme liberalism/left] to 10=[Extreme conservativism/right]), completed a baseline measure of affect towards climate action, and completed the Cognitive Reflection Task, Version 2, in random order. A two-strikes-out attention check ([Please select "3" to make sure you are paying attention]) was presented; failing it triggered a warning with a 10-seconds time penalty. Inattentive participants received the attention check for a second time, and participants found inattentive again afterwards were screened out of the survey (n=16). Attentive participants were then randomly allocated to one of the eight conditions and received the assigned intervention or, for participants in the passive disinformation control condition, were directly forwarded to the following section of the experiment. Participants in the pure control condition received neither the interventions nor the climate disinformation statements. All interventions were presented sequentially in four screens, with a 5-20s time lock (depending on the content length of each screen) that did not allow participants to manually proceed to the next screen until the time had elapsed. A manipulation check measuring participants' motivation to resist persuasion followed. Afterwards, participants received twenty real climate disinformation statements in form of anonymous tweets, in randomized order with a 2s time lock, and report their affect towards actions to mitigate climate change after each disinformation statement. Following the disinformation provision, participants completed the climate change perceptions scale, a modified version of the Working for Environmental Protection Task, and the truth discernment task, all described in full detail below. Finally, we probed participant's understanding of the experimental aims with an open-ended question to account for potential demand effects. The survey ended with an extended debriefing that contained a reminder of the scientific consensus behind climate change with a link to the latest IPCC report. Survey duration was about 21 minutes. |
| Timing | Data collection started the 15th of May, 2023, and terminated on the 27th of May, 2023. |
| Data exclusions | No participant was excluded from data analysis. |
| Non-participation | In total, n = 1171 participants dropped out/declined participation (USA n = 113, Canada n = 94, UK n = 143, Ireland n = 113, Australia n = 104, New Zealand n = 66, Singapore n = 58, Philippines n = 109, India n = 94, Pakistan n = 128, Nigeria n = 6, and South Africa n = 143). |
| Randomization | Participants were randomly assigned to one of eight different between-participants conditions: pure control (no inoculation, no disinformation), passive disinformation control (disinformation without inoculation), scientific consensus inoculation, trust in scientists inoculation, transparent communication inoculation, moralization of climate action inoculation, accuracy inoculation, and positive emotion inoculation. |

# Reporting for specific materials, systems and methods

We require information from authors about some types of materials, experimental systems and methods used in many studies. Here, indicate whether each material, system or method listed is relevant to your study. If you are not sure if a list item applies to your research, read the appropriate section before selecting a response.

## Materials & experimental systems

| n/a | Involved in the study | |
|---|---|---|
| ☒ | ☐ | Antibodies |
| ☒ | ☐ | Eukaryotic cell lines |
| ☒ | ☐ | Palaeontology and archaeology |
| ☒ | ☐ | Animals and other organisms |
| ☒ | ☐ | Clinical data |
| ☒ | ☐ | Dual use research of concern |
| ☒ | ☐ | Plants |

## Methods

| n/a | Involved in the study | |
|---|---|---|
| ☒ | ☐ | ChIP-seq |
| ☒ | ☐ | Flow cytometry |
| ☒ | ☐ | MRI-based neuroimaging |
